# Muscle Thickness and Curvature Influence Atrial Conduction Velocities

**DOI:** 10.3389/fphys.2018.01344

**Published:** 2018-10-29

**Authors:** Simone Rossi, Stephen Gaeta, Boyce E. Griffith, Craig S. Henriquez

**Affiliations:** ^1^Cardiovascular Modeling and Simulation Laboratory, Carolina Center for Interdisciplinary Applied Mathematics, University of North Carolina, Chapel Hill, NC, United States; ^2^Clinical Cardiac Electrophysiology/Cardiology Division, Duke University Medical Center, Durham, NC, United States; ^3^Departments of Mathematics, Applied Physical Sciences, and Biomedical Engineering, University of North Carolina, Chapel Hill, NC, United States; ^4^McAllister Heart Institute, University of North Carolina, Chapel Hill, NC, United States; ^5^Department of Biomedical Engineering, Pratt School of Engineering, Duke University, Durham, NC, United States

**Keywords:** cardiac electrophysiology, bidomain model, conduction velocity, bath-loading conditions, left atrial posterior wall, electroanatomical mapping, atrial fibrillation

## Abstract

Electroanatomical mapping is currently used to provide clinicians with information about the electrophysiological state of the heart and to guide interventions like ablation. These maps can be used to identify ectopic triggers of an arrhythmia such as atrial fibrillation (AF) or changes in the conduction velocity (CV) that have been associated with poor cell to cell coupling or fibrosis. Unfortunately, many factors are known to affect CV, including membrane excitability, pacing rate, wavefront curvature, and bath loading, making interpretation challenging. In this work, we show how endocardial conduction velocities are also affected by the geometrical factors of muscle thickness and wall curvature. Using an idealized three-dimensional strand, we show that transverse conductivities and boundary conditions can slow down or speed up signal propagation, depending on the curvature of the muscle tissue. In fact, a planar wavefront that is parallel to a straight line normal to the mid-surface does not remain normal to the mid-surface in a curved domain. We further demonstrate that the conclusions drawn from the idealized test case can be used to explain spatial changes in conduction velocities in a patient-specific reconstruction of the left atrial posterior wall. The simulations suggest that the widespread assumption of treating atrial muscle as a two-dimensional manifold for electrophysiological simulations will not accurately represent the endocardial conduction velocities in regions of the heart thicker than 0.5 mm with significant wall curvature.

## 1. Introduction

Atrial fibrillation (AFib) is the most common cardiac arrhythmia, and symptoms can range from being nonexistent to severe, possibly leading to stroke, heart failure, sudden death, and cardiovascular morbidity (January et al., 2014; Kirchhof et al., 2016). Electroanatomic mapping, which involves acquiring extracellular signals (electrograms) at multiple locations using catheter-based electrode, is often used in clinical procedures to identify triggers of the AF and to characterize the electrophysiological health of the tissue. One outcome of this mapping is a display of the pattern of the spread electrical activation obtained by identifying the local activation time from the electrograms. These activation maps can be used to estimate the conduction velocity and help to localize regions of slow conduction associated with cellular decoupling and fibrosis (King et al., 2013; Grossi et al., 2016). Several approaches can be used to evaluate CVs from the measured electrophysiological data, such as polynomial surface-fitting algorithms, finite-difference techniques, and triangulation, among many others (Cantwell et al., 2015). Because of the paucity of data that can be acquired at high resolution in a clinical procedure, accurate CV estimates are difficult to obtain, particularly in regions of the heart with significant curvature. In addition, it is well known that conduction velocity is very sensitive to membrane excitability, tissue conductivity, fiber orientation, wavefront shape, and even the properties of the adjoining blood, making interpretation of CV measurements challenging at best.

To better understand the various factors affecting both normal and abnormal conduction, computer models of the atria have been developed (Harrild and Henriquez, 2000; Seemann et al., 2006; Muñoz et al., 2011; McDowell et al., 2012; Rossi and Griffith, 2017). Because of the high computational cost required by these simulations, the atria are sometimes simplified as a single two-dimensional manifold (Harrild and Henriquez, 2000; Seemann et al., 2006; Muñoz et al., 2011; McDowell et al., 2012; Rossi and Griffith, 2017). However, this surface representation of the atria cannot be used to describe the endo-epicardial electrical dissociation taking place during AFib (Gharaviri et al., 2012). To overcome this limitation, bilayer models have been proposed (Gharaviri et al., 2012; Labarthe et al., 2014; Coudière et al., 2017). Although these models have a reduced computational cost with respect to fully three-dimensional simulations, they fail to capture the loading of the muscle thickness and adjoining blood layer. The complete effects of the geometric factors on conduction can only be determined in a volumetric model of the atria. The goal of this work is to investigate how wall thickness and curvature affect conduction velocity and whether these geometric factors need to be considered in modeling relatively thin tissue such as the atria. In addition, we investigate how the thickness of the adjoining blood layer affects the CV and the resulting extracellular signals. Simulations are performed on idealized geometries and a patient specific geometry of the posterior wall of the atria. The results show that variations of more than 10% in CV can derive from the atrial geometry even without considering changes in the transmural properties.

The blood is the natural volume conductor that bathes the cardiac wall (Trayanova, 1997). Since endocardial bipolar signals measured by electroanatomical mapping systems are influenced by the presence of blood, our computational model is augmented with a perfusing endocardial bath (Henriquez et al., 1996). The role of muscle thickness on the CV in the presence of a bath has been studied only on a thick strand of muscle without curvature (Roth, 1991). Although the role of the perfusing bath has been extensively studied (Roth, 1991, 1996; Henriquez et al., 1996; Trayanova, 1997; Srinivasan and Roth, 2004; Vigmond et al., 2009; Bishop et al., 2011; Colli-Franzone et al., 2011), and methods have been proposed to reduce the computational demands of these simulations (Bishop and Plank, 2011), the minimum depth of the bath that adequately accounts for the bath-loading conditions on CV is not currently known. For this reason, we investigate the role of the bath thickness on the CVs and bipolar signals. Our results show that a bath thickness of at least 1.5 mm is needed to capture endocardial CVs with good accuracy. The same thickness of the intracardiac bath layer also guarantees a satisfactory representation of the endocardial bipolar signals.

## 2. The bidomain model

Most common tissue-scale models of cardiac electrophysiology consider the myocardium to be composed of continuous intracellular and extracellular compartments, coupled via a continuous cellular membrane. This study considers such a model; specifically, we consider the bidomain model of the propagation of the action potential in cardiac muscle. The bidomain model of the propagation of the action potential in cardiac muscle formulated by Tung (1978) is a continuum model derived from a homogenized description of excitation propagation in the cardiac microarchitecture (Neu and Krassowska, 1993; Keener and Sneyd, 1998; Griffith and Peskin, 2013).

The bidomain equations describe the dynamics of a local average of the voltages in the intracellular and extracellular compartments over a control volume. One of the assumptions required by the homogenization procedure is that the control volume is large compared to the scale of the cellular micro-architecture, but small compared to any other important spatial scale of the system, such as the width of the action potential wavefront. Although the validity of this model has been questioned, for example by Bueno-Orovio et al. (2014), this approach has been extremely successful, and at present, most organ-scale simulations of cardiac electrophysiology use such a model. For a detailed review of the bidomain model and other models of cardiac electrophysiology, we refer to other references (Griffith and Peskin, 2013; Franzone et al., 2014).

**Figure 1:**
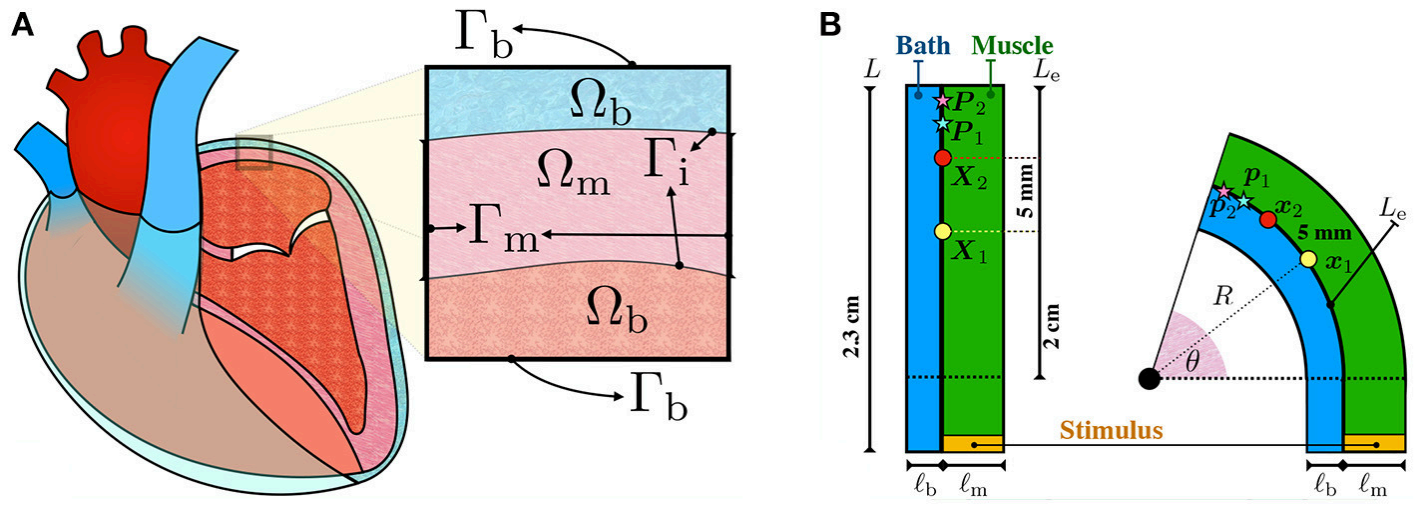
**(A)** Schematic representation of the configurations of the muscle and blood bath. Inside the heart, blood acts as a low resistance conductor. Outside the heart, between the epicardium and the epicardial sac, an interstitial fluid can also act as a conductor. **(B)** Schematic representation of the idealized left atrial posterior wall. A rectangular strand of muscle (green) of length *L* = 2.3 cm and thickness ℓ_m_ is adjacent to an endocardial bath (blue) of thickness ℓ_b_. The thickness of the muscle ℓ_m_ and of the bath ℓ_b_ are varied to study their influence on endocardial CVs. Curvature is applied to the top part of the rectangle such that the curved endocardial length *L*_e_ is fixed at 2 cm. The corresponding curvature κ is defined as the inverse of the endocardial radius. The curvature is positive if the muscle is bent to the left and it is negative if it is bent to the right. Endocardial CVs are measured using the activation times at ***x***_1_ (yellow circle) and ***x***_2_ (red circle). In the straight geometry, ***x***_1_ and ***x***_2_ correspond to the points **X**_1_=(0 cm, 1 cm) and **X**_2_=(0 cm, 1.5 cm), fixed at distance 5 mm. As described by equation (7), endocardial CVs are defined as the distance between these two points divided by the difference of the respective activation times. Unipolar extracellular signals ${V_{\text{e}}}^{1}$ and ${V_{\text{e}}}^{2}$ are recorded at 1 kHz at ***p***_1_ and ***p***_2_, corresponding to the points **P**_1_=(0 cm, 1.75 cm) and **P**_2_=(0 cm, 1.95 cm) in the straight geometry. Bipolar signals were computed as the difference ${V_{\text{e}}}^{2}-{V_{\text{e}}}^{1}$.

In our model, we also consider a conductive blood cavity adjacent to the tissue. In the bidomain model, current flow is restricted to the intracellular (denoted by subscript i), extracellular (denoted by subscript e), and bath (denoted by subscript b) compartments and is described by a set of coupled partial differential equations. Referring to Figure 1A, Ω_m_ denotes the muscle region and Ω_b_ denotes the perfusing bath. From charge conservation, the bidomain equations can be written in the muscle region Ω_m_ as

(1) $$\begin{array}{l} \nabla \cdot \left(D_{\text{i}}\nabla V\right)+\nabla \cdot \left(D_{\text{i}}\nabla V_{\text{e}}\right)=\chi \left(C_{\text{m}}\partial_{t}V+I_{\text{ion}}\right)-I_{\text{i}}^{\text{v}}, \end{array}$$

(2) $$\begin{array}{l} \nabla \cdot \left(D_{\text{i}}\nabla V\right)+\nabla \cdot \left(\left(D_{\text{i}}+D_{\text{e}}\right)\nabla V_{\text{e}}\right)=I_{\text{total}}^{\text{v}}, \end{array}$$

in which *V*_i_ and *V*_e_ are the potentials of the homogenized intracellular and extracellular compartments, respectively, such that *V* = *V*_i_ − *V*_e_ is the transmembrane potential difference, ***D***_i_ and ***D***_e_ are the intracellular and extracellular conductivity tensors, *C*_m_ is the membrane capacitance, χ is membrane area per unit volume of tissue, and $I_{\text{i}}^{\text{v}}$ and $I_{\text{e}}^{\text{v}}$ are the volumetric intracellular and extracellular applied currents such that $I_{\text{total}}^{\text{v}}=I_{\text{i}}^{\text{v}}+I_{\text{e}}^{\text{v}}$. The dynamics of the transmembrane current *I*_ion_, accounting for charged ionic species moving from the intracellular compartment to the extracellular compartment and vice-versa, are described by a set of ordinary differential equations, called the ionic model. More precisely, the ionic model introduces the additional variables ***w***, satisfying ∂_*t*_***w*** = ***g***(*V*, ***w***), such that *I*_ion_ = *I*_ion_(*V*, ***w***).

In the blood region, the bath potential *V*_b_ satisfies the Poisson's equation

(3) $$\begin{array}{l} \nabla \cdot \left(D_{\text{b}}\nabla V_{\text{b}}\right)=I_{\text{b}}, \end{array}$$

in which ***D***_b_ represents the blood conductivity tensor and *I*_b_ is a volumetric applied current in the blood domain.

The anisotropic nature of the muscle is accounted for in the bidomain model through the conductivity tensors ***D***_i_ and ***D***_e_. Denoting with **f** the local direction of the fiber field, we assume axial symmetry relative to **f**, such that the conductivity tensors can be written as $D_{\text{i}}=\sigma_{\text{i}}^{\text{t}}I+\left(\sigma_{\text{i}}^{\text{f}}-\sigma_{\text{i}}^{\text{t}}\right)\text{f}⊗\text{f}$, and $D_{\text{e}}=\sigma_{\text{e}}^{\text{t}}I+\left(\sigma_{\text{e}}^{\text{f}}-\sigma_{\text{e}}^{\text{t}}\right)\text{f}⊗\text{f}$. Here, $\sigma_{\text{i}}^{\text{f}}$ and $\sigma_{\text{i}}^{\text{t}}$ denote the tissue conductivities along and across the fiber direction in the intracellular space, $\sigma_{\text{e}}^{\text{f}}$ and $\sigma_{\text{e}}^{\text{t}}$ denote the extracellular conductivities, and ***I*** is the identity tensor. The blood conductivity is assumed isotropic, so that ***D***_b_ = σ_b_***I***. Representative values of the intracellular, extracellular, and blood conductivities are taken from the work of Roth (1996). Specifically, we set $\sigma_{\text{e}}^{\text{f}}=\sigma_{\text{i}}^{\text{f}}=4.5$ mS/cm, $\sigma_{\text{e}}^{\text{t}}=1.8$ mS/cm, $\sigma_{\text{i}}^{\text{t}}=0.45$ mS/cm, and σ_b_ = 20 mS/cm.

The boundary conditions for the tissue are those derived for a spatially periodic cellular syncytium by Krassowska and Neu (1994). Referring to Figure 1A, at the muscle boundaries Γ_m_, current fluxes in the intracellular and extracellular compartments depend on externally applied surface currents,

(4) $$\begin{array}{ll} \text{n}\cdot \left(D_{\text{i}}\nabla V_{\text{i}}\right) & =\text{n}\cdot \left(D_{\text{i}}\nabla \left(V+V_{\text{e}}\right)\right)=I_{\text{i}}^{\text{s}}, \end{array}$$

(5) $$\begin{array}{ll} \text{n}\cdot \left(D_{\text{e}}\nabla V_{\text{e}}\right) & =I_{\text{e}}^{\text{s}}, \end{array}$$

in which the vector **n** is the outward unit normal to the boundary of the tissue domain. In the following, we shall assume that the intracellular and extracellular surface currents, $I_{\text{i}}^{\text{s}}$ and $I_{\text{e}}^{\text{s}}$, are zero. At the bath boundary Γ_b_, we enforce no current flux in the blood domain **n** · (***D***_b_ ∇ *V*_b_) = 0. Along the muscle-blood interface Γ_i_, we require continuity of the extracellular and bath potentials *V*_e_ = *V*_b_, continuity of the normal currents **n** · (***D***_e_ ∇ *V*_e_) = **n** · (***D***_b_ ∇ *V*_b_) and zero intracellular current density **n** · (***D***_i_ ∇ *V*_i_). We refer to the Supplementary Material for more details on the model and its numerical discretization.

A modified version of the Courtemanche et al. (1998) ionic model available on Model DB (Carnevale and Hines, 2006; McDougal et al., 2017), defining the ionic current *I*_ion_ and gating variable dynamics ***g***(*V*, ***w***), is chosen to represent human atrial action potential.

### 2.1. Idealized model of the atrial left posterior wall

To study the effects of muscle thickness, muscle curvature, and bath-loading conditions on the measured conduction velocities, we devised a simple idealized test case. Although our main interest is to understand measurements of endocardial CVs in the posterior wall of the left atrium, which has overall positive curvature, our study is not limited to that application. For this reason, we also consider negative curvatures. Although those cases are not representative of the left atrial posterior wall, the relationships between negative curvature and CV can be easily explored in our idealized model.

A strand of muscle is connected to a bath as depicted in Figure 1B. This simplified model represents a piece of atrial tissue where the endocardial surface Γ_i_ separates the blood from the muscle. We consider a straight strand of tissue of length 2.3 cm. To study the influence of curvature, the strand of tissue is then bent on both sides keeping the length of the endocardial interface fixed. The epicardial boundary is instead allowed to change in length. For this reason, curved domains with opposite curvature are not symmetric.

Consider Figure 1B. The initial muscle domain is a rectangle of length *L* = 2.3 cm and thickness ℓ_m_, such that Ω_m_ = [0 cm, ℓ_m_] × [−0.3 cm, 2 cm]. Denote with *L*_e_=2 cm the length of the rectangle where curvature will be varied. We set the “endocardial” surface Γ_i_ = {0 cm} × [−0.3 cm, *L*_e_] on the left edge of the muscle. The length *L*_e_ = 2 cm denotes the length of the endocardial surface where curvature will be imposed. A bath is added adjacent to the interface Γ_i_ with thickness ℓ_b_, such that Ω_b_ = [−ℓ_b_, 0 cm] × [−0.3 cm, *L*_e_]. These geometrical settings represent the straight domain with zero curvature. We denote with (*X, Y*) the coordinates of a point in this straight domain. Given an angle θ ∈ (0, π], we bend the domain, keeping the measure *L*_e_ of the top part of the endocardial interface Γ_i_ fixed at 2 cm. The transformation from the straight rectangle to the curved one is performed using the relations

(6) $$\begin{array}{ll} x & =\{\begin{array}{cc} X & \text{if }Y\leq 0,\\ c\left(R_{\text{0}}-R\cos\left(\theta \frac{Y}{Y_{\text{max}}}\right)\right) & \text{if }Y>0, \end{array}\\ y & =\{\begin{array}{cc} Y & \text{if }Y\leq 0,\\ cR\sin\left(\theta \frac{Y}{Y_{\text{max}}}\right) & \text{if }Y>0, \end{array} \end{array}$$

in which the parameter *c* specifies in which direction the bending is performed, that is, *c* = −1 means bending to the right whereas *c* = 1 means bending to the left. The radius of curvature of Γ_i_ is given by *R*_0_ = *Y*_max_/θ, such that *R* = *R*_0_ + *cX*, in which *Y*_max_ is the maximum *Y* coordinate. We define the curvature of the interface Γ_i_ by κ = 1/(*cR*_0_), such that the curvature is negative if *c* is negative (bend to the right) and positive if *c* is positive (bend to the left). Given such construction, geometries with opposite curvature will not be symmetric even though the length and the magnitude of the curvature of the endocardial interface are the same. For any possible curvature, the region defined by the points {(*x, y*) ∈ ℝ^3^:*y* ≤ 0} is the same in all cases. Applying the same initial conditions and the same initial stimulus in this region, we can compare the effects of curvature on the conduction velocity.

Before applying any curvature, the domain Ω is discretized using a structured triangular mesh with mesh size *h*_*Y*_ = 50 μm in the longitudinal fiber direction and *h*_*X*_ = 25 μm in the transversal fiber direction. Denoting with *v*_*X*_ the conduction velocity in the longitudinal fiber direction, we used the CFL condition *h*_*X*_/*v*_*X*_ ≤ 1 to determine the timestep (Rossi and Griffith, 2017). We chose the largest negative power of 2 such that the CFL condition was satisfied, which led to the timestep Δ*t* = 0.03125 ms. This choice is also sufficient to ensure the stability of the time integrator used for the ionic model.

To quantify the changes in endocardial conduction velocity with respect to the curvature, we use a simple finite difference method: measuring the activation times *t*_1_ and *t*_2_ on the endocardial surface Γ_i_ at two locations ***x***_1_ and ***x***_2_, corresponding to the points ***X***_1_ = (0 cm, 1 cm) and ***X***_2_ = (0 cm, 1.5 cm) in the straight domain (no curvature), we define the conduction velocity as

(7) $$\begin{array}{l} v=\frac{∥x_{2}-x_{1}{∥}_{\Gamma_{\text{i}}}}{t_{2}-t_{1}}=\frac{∥X_{2}-X_{1}∥}{t_{2}-t_{1}}=\frac{0.5\text{cm}}{t_{2}-t_{1}}, \end{array}$$

where ∥ · ∥_Γ_i__ represents the distance between ***x***_1_ and ***x***_2_ on the endocarial surface. Referring to Figure 1B, the points ***x***_1_ and ***x***_2_ correspond to the position on the muscle-bath interface Γ_i_ of the yellow and red circles, respectively.

An equal and opposite stimulus is applied in the interior and exterior compartments of the muscles such that $I_{\text{e}}^{\text{v}}=100\;\mu {\text{A/cm}}^{3}$ for the first 2 ms if *y* was smaller than –0.2797 mm. This choice generates a plane wave propagating in the longitudinal direction whenever the domain is straight (no curvature) and no bath region is considered.

Unipolar signals ${V_{\text{e}}}^{1}$ and ${V_{\text{e}}}^{2}$ were recorded at 1 kHz on the endocardial surface at ***p***_1_ and ***p***_2_ corresponding to the points ***P***_1_ = (0 cm, 1.75 cm) and ***P***_2_ = (0 cm, 1.95 cm) of the straight domain. Referring to Figure 1B, the points ***p***_1_ and ***p***_2_ correspond to the position on the muscle-bath interface Γ_i_ of the light blue and pink red stars, respectively. Bipolar signals were then computed taking the difference ${V_{\text{e}}}^{2}-{V_{\text{e}}}^{1}$.

### 2.2. Left atrial posterior wall

A detailed geometry of the whole left atrium was collected by fast anatomical mapping (FAM) with a 2-5-2 PentaRay catheter. High-density maps of the left atrial posterior wall (LAPW) endocardial surface were created using the Carto3 electroanatomic mapping system (Biosense Webster, Diamond Bar, CA). The LAPW was mapped following pulmonary vein isolation by wide antral circumferential ablation (WACA). The region was defined as the area of the posterior left atrium between WACA lesion sets encircling the bilateral pulmonary veins and extending from their inferior margin to their superior margin. The LAPW surface was extracted from the reconstructed geometry of the left atrium and its geometrical representation was generated using SOLIDWORKS (Dassault Systèmes, Waltham, MA). The surface was then thickened outward to obtain a uniform 1.5 mm LAPW thickness. The bath region was created thickening in the opposite direction for 2.85 mm. We used the Trelis software (Computational Simulation Software, American Fork, UT American Fork, Utah) to generate a simplex mesh of the LAPW with bath. The mesh size for muscle domain was selected to yield 16 elements through the muscle thickness. As the solution in the bath is smooth, a larger mesh size was used in the bath domain. Still, on the muscle-bath interface Γ_i_, the two meshes are conforming.

**Figure 2:**
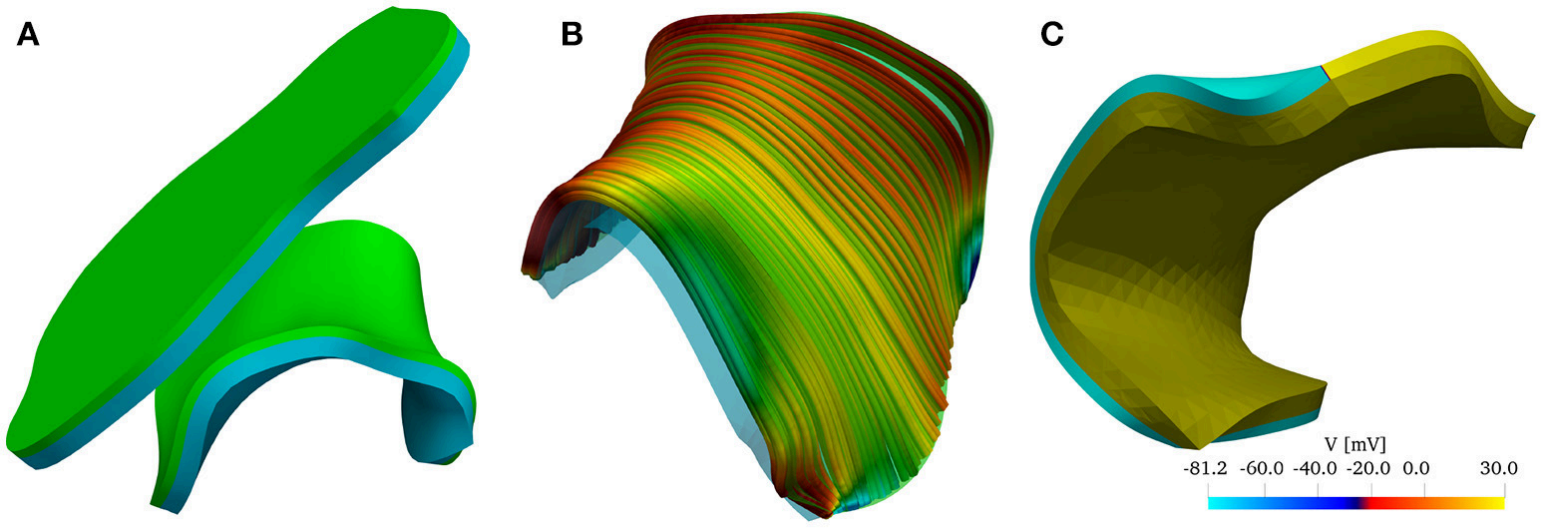
**(A)** Reconstructed model of the left atrial posterior wall (LAPW) and its flattened representation. The muscle domain of thickness 1.5 mm is represented in green, and the intracardiac bath of thickness 2.85 mm is presented in light blue. **(B)** Fiber field of the posterior wall generated by a rule-based method based on Poisson interpolation. The fibers fields are defined only in the muscular region of the computational domain. The color scheme, representing the *z*-component of the fiber vector field, is chosen to highlight the direction in which the muscle fibers are aligned. **(C)** Graphical representation of the initial condition of the simulation: an intracellular potential of 30 mV is set at the bottom edge of the left atrial posterior wall. In the bath the transmembrane potential is not defined, and the whole bath is represented in dark yellow.

To investigate the role of muscle thickness and curvature on the LAPW, we used SOLIDWORKS to flatten the LAPW endocardial surface. The same procedure as for the curved LAPW was then used to thicken and mesh the resulting geometry. The resulting geometries are shown in Figure 2A.

The fiber field was created by assuming the existence of a harmonic potential φ(***x***) such that **f** = ∇φ. In practice, we solve numerically the equation Δφ = 0 with mixed boundary conditions. In particular, we set φ = 0 on the surface Γ_0_ and φ = 1 on Γ_1_, and ∂_**n**_φ = 0 on ∂Ω_m_\(Γ_0_ ∪ Γ_1_), where the surfaces Γ_0_, and Γ_1_ are the boundaries delimiting the LAPW from the top and from the bottom. The resulting fiber field, depicted in Figure 2B, qualitatively matches the anatomical structures of the LAPW shown by Markides et al. (2003). Referring to Figure 2B, Γ_0_ and Γ_1_ are the boundaries orthogonal to the fiber field. Similarly, a fiber field was generated for the flattened geometry.

The simulations were initiated by imposing an initial condition for the transmembrane potential *V*. As shown in Figure 2C, the potential was set to 30 mV on Γ_0_ and to its resting value of −81.2 mV everywhere else. Similarly, we imposed the initial condition on the flattened geometry.

For both the LAPW and the flattened LAPW, we solved the bidomain equations in three scenarios: (1) considering only the endocardial surface (referred to as 2D); (2) considering only the muscle domain (referred to as 3D); and (3) considering the muscle with bath-loading conditions (referred to as Bath). In all cases, we registered the activation times *A*_t_(***x***), as the earliest time when the transmembrane potential was larger than −5 mV. The endocardial conduction velocities were then reconstructed on each node of the triangulation in the following way. For each triangle *K* on the endocardial surface, we define the elemental conduction velocity ***v***_*K*_ = ∇*A*_t_/(∇*A*_t_ · ∇*A*_t_). Since *A*_t_ is interpolated between the nodes using linear basis functions on each element, its gradient ∇*A*_t_ and the conduction velocity ***v***_*K*_ are constant on each triangle. For each vertex *q*, we define the patch Π_*q*_ as the set of triangles *K* surrounding the node *q*. The averaged nodal velocity is then given as

(8) $$v_{q}=\frac{\sum_{K\in \prod_{q}}\left|K\right|v_{K}}{\sum_{K\in \prod_{q}}\left|K\right|},$$

in which |*K*| denotes the area of the triangle *K*.

## 3. Numerical results

The bidomain model was discretized in space using linear finite elements (Plank et al., 2005; Franzone et al., 2006; Pathmanathan et al., 2010; Bishop and Plank, 2011; Landajuela et al., 2018). The intracellular, extracellular, and bath potentials are solved monolithically (Bernabeu and Kay, 2011), using IMEX temporal schemes. We use the C++ implementation of the model of Courtemanche et al. (1998) provided by Hsing-Jung Lai and Sheng-Nan Wu on Model DB (McDougal et al., 2017), which includes the modifications by Ingemar Jacobson (Carnevale and Hines, 2006) needed for ion concentrations to be stable at a pacing rate of 1 Hz. Since the Courtemanche ionic model contains many discontinuous parameters that negatively influence the expected optimal rate of convergence of the finite element discretization (Arthurs et al., 2012), we rely on the simple IMEX BDF1 method. We refer to the Supplementary Material for more details on the numerical methods used. Unless explicitly stated, we used the same set of parameters for all the numerical tests presented below. The parameters are reported in Table 1.

**Table 1:** Bidomain model parameters used in the numerical simulations.

**$\sigma_{\text{e}}^{\text{f}}\left[\text{mS}{\text{cm}}^{-1}\right]$**	**$\sigma_{\text{e}}^{\text{t}}\left[\text{mS}{\text{cm}}^{-1}\right]$**	**$\sigma_{\text{i}}^{\text{f}}\left[\text{mS}{\text{cm}}^{-1}\right]$**	**$\sigma_{\text{i}}^{\text{t}}\left[\text{mS}{\text{cm}}^{-1}\right]$**	**$\sigma_{\text{b}}\left[\text{mS}{\text{cm}}^{-1}\right]$**	**${\text{C}}_{\text{m}}\left[\mu \text{F}{\text{cm}}^{-2}\right]$**	****χ[cm^−1^]****
4.5	1.8	4.5	0.45	20	1	1000

The code developed in this work, BeatIt (available at github.com/rossisimone/beatit), relies on the parallel C++ finite element library libMesh (Kirk et al., 2006) and on PETSc (Balay et al., 1997, 2017) and HYPRE linear solvers (Falgout and Yang, 2002). More specifically, we used the FieldSplit preconditioner (Brown et al., 2012) provided by PETSc to solve the system using the block Gauss-Seidel method, and each sub-block is preconditioned using BoomerAMG (Falgout et al., 2010). More details about the algorithmic implementation can be found in the Supplementary Material. Using a uniform structured grid for the muscle and bath domain, simulations of the two-dimensional idealized test case were run in serial on a Linux workstation. Simulations on the patient-specific left atrial posterior wall used a fine discretization in the muscle domain and a coarse one in the bath domain. A boundary layer in the mesh of the bath was created to correctly resolve the bath potential close to the muscle interface. Simulations were run on a single node (44 processors) of the Dogwood Linux cluster at the University of North Carolina at Chapel Hill. The visualization of the results and their analysis have been carried out using Paraview (Ahrens et al., 2005) and MATLAB The Mathworks, Inc., Natick, MA.

### 3.1. Without bath-loading conditions endocardial CVs depend on tissue-thickness and curvature

We start investigating how muscle thickness influences the endocardial conduction velocities when no bath-loading conditions are considered. For this test, we consider muscles of thicknesses ℓ_m_ = 0.025, 0.5, 1, 1.5, and 2 mm. The thickness ℓ_m_ = 0.025 mm corresponds to the case where the atrial tissue is considered to be so thin that can be approximated with a bidimensional manifold.

**Figure 3:**
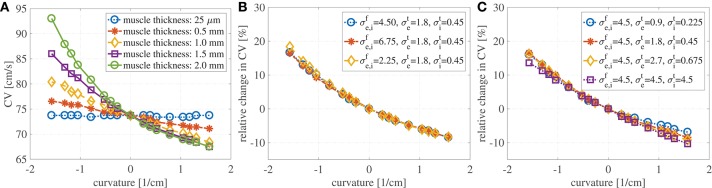
**(A)** Endocardial CVs as a function of the curvature when no bath-loading conditions are considered (ℓ_b_ = 0 mm) for muscle thicknesses ℓ_bm_ = 0.25 μm, 0.5, 1, 1.5, and 2 mm. Note that the construction of the domain leads to different geometries for positive and negative curvatures. This is because we keep the endocardial length fixed, but we allow the epicardial surface to become shorter or longer. When a planar wave travels on a curved domain, endocardial CV are faster for negative curvatures and slower for positive curvatures. Since the left atrial posterior wall has mostly positive curvature, endocardial CVs are expected to be slower than in a flat piece of muscle. The case of mucles thickness 25 μm correspends to the case of two-dimensional manifolds in three-dimensional simulations. **(B)** Relative change in conduction velocities (with respect to CVs in a straight muscle) as a function of curvature for three selected values of the longitudinal conductivity coefficients but fixed transveral conductivities. Muscle thickness was fixed at 1.5 mm. Relative changes in CVs are not influenced by longitudinal conductivities. **(C)** Relative change in conduction velocities (with respect to CVs in a straight muscle) as a function of curvature for four selected values of the transversal conductivity coefficients but fixed longitudinal conductivities. Muscle thickness was fixed at 1.5 mm. Even for isotropic conditions (purple) endocardial CVs change depending on the curvature. Relative changes in CVs are only slightly influenced by transversal conductivities.

Figure 3A shows the evaluation of the conduction velocities on the endocardial surface for the considered muscle thicknesses. When the curvature is zero, the conduction velocity is independent of muscle thickness. If the muscle thickness is small enough, say of the order of a handful of cardiomyocytes, the conduction velocities are also independent of the curvature. On the other hand, when curvature is imposed on a thicker muscle the endocardial conduction velocities can change quite drastically. For positive curvatures (bending to the left) the endocardial CVs become slower, while for negative curvatures (bending to the right) the endocardial CVs become faster.

We also test if the relative changes in the CVs are influenced by the anisotropy ratio (AR) for muscle thickness of 1.5 mm. In a first test we have increased and decreased the longitudinal conductivities $\sigma_{\text{e}}^{\text{f}}=\sigma_{\text{i}}^{\text{f}}$ by 50%, keeping fixed the transversal conductivities. As shown in Figure 3B, variations in the longitudinal conductivities do not affect the relative changes in CVs with respect to curvature. Clearly, the magnitude of the CVs is different. For κ = 0 cm^−1^, if $\sigma_{\text{e}}^{\text{f}}=\sigma_{\text{i}}^{\text{f}}=$ 4.5 mS/cm, the CV is measured to be 73.7 cm/s; if $\sigma_{\text{e}}^{\text{f}}=\sigma_{\text{i}}^{\text{f}}=$ 6.75 mS/cm (+50% case), the CV is measured to be 90.4 cm/s; if $\sigma_{\text{e}}^{\text{f}}=\sigma_{\text{i}}^{\text{f}}=$ 2.25 mS/cm (–50% case), the CV is measured to be 51.8 cm/s. These values are in accordance with the expected dependence on the CVs on the square root of the conductivities. In a second test, we have increased and decreased the transversal conductivities $\sigma_{\text{e}}^{\text{f}}=\sigma_{\text{i}}^{\text{f}}$ by 50%, keeping fixed the longitudinal conductivities. The relative changes in CVs are shown in Figure 3C, where we have also included the results for isotropic propagation. Although some differences can be found at different anisotropy ratios, changes in the ARs seem to only have a minor effect on the relative changes in CVs. In all these cases for which κ = 0 cm^−1^, the endocardial CV was measured to be about 73.7 cm/s.

**Figure 4:**
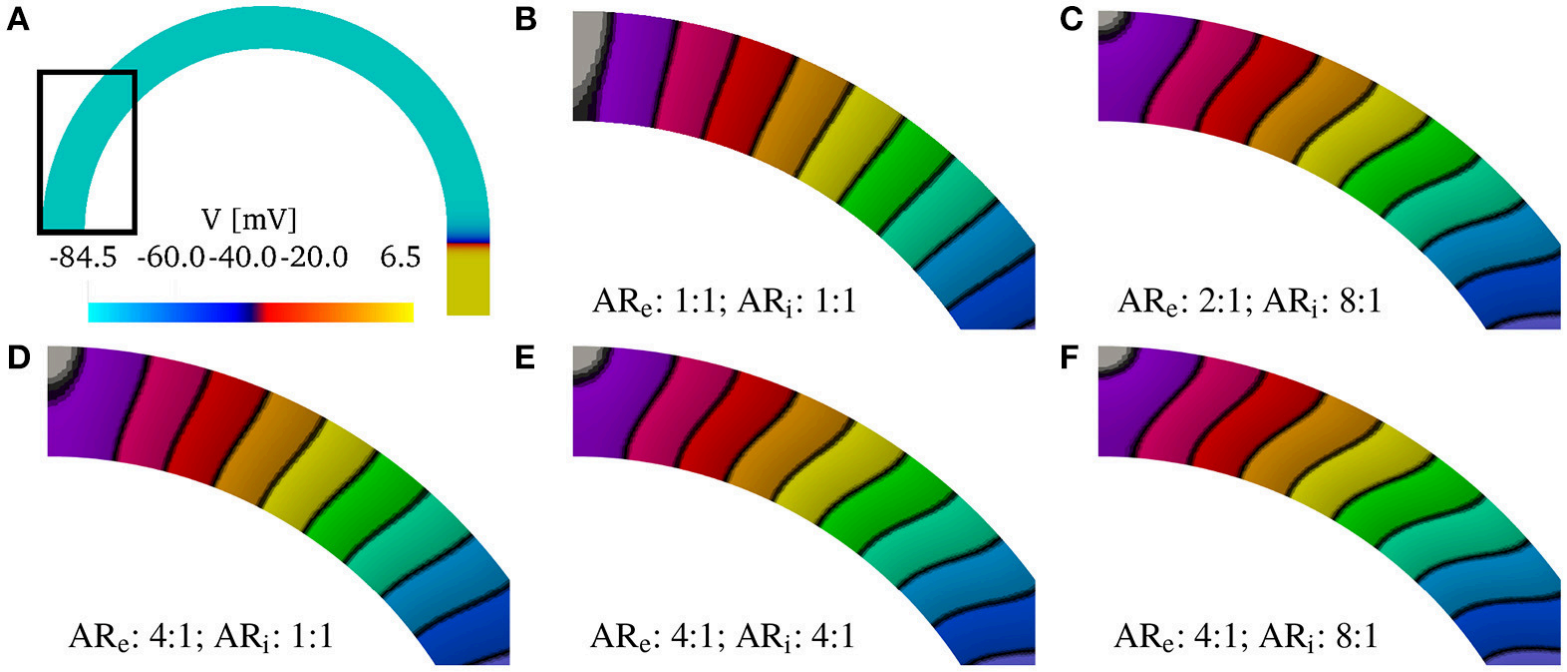
**(A)** Transmembrane potential wavefront at *t* = 4 ms without bath-loading conditions for curvature κ = π/2 cm^−1^. The initial stimulus generates a plane wave in the straight part of the muscle for any anisotropy ratio (AR). **(B–F)** Shape of the wavefronts at 1ms distance (zoom of the rectangular region in ***A***) for several external (AR_e_) and internal (AR_i_) anisotropy ratios. The longitudinal conductivity $\sigma_{\text{e}}^{\text{f}}=\sigma_{\text{i}}^{\text{f}}$ is fixed for all cases and the transversal condcitivities are changed. While in the isotropic case **(B)** the fronts remain almost planar, in all other cases, the wavefronts become curved.

Although, we have found that the AR does not influence the relative changes in the CVs, AR does influence the shape of the wavefront in the curved domains. We show in Figure 4 the shapes of the wavefronts at different internal and external anisotropic ratios (AR_i_ and AR_i_) for κ = π/2. Specifically, fixing the longitudinal conductivity coefficients $\sigma_{\text{e}}^{\text{f}}=\sigma_{\text{i}}^{\text{f}}=$ 4.5 mS/cm, we show the activation times (black lines are iscochrones separated by 1 ms increments) in various cases changing the transversal conductivities. As shown in Figure 4A, the initial condition creates a plane wave in the straight region of the domain. Figure 4A shows the rectangular region where we look at the wavefronts. Under isotropic conditions, the wavefronts remain straight even in the curved domain. This is shown in Figure 4B, where the isochrones are radial. Under anisotropic conditions, Figures 4C–F, the wavefronts have a different orientation with respect to the radial direction. Additionally, the boundary conditions induce wavefront curvatures close to the boundaries.

**Figure 5:**
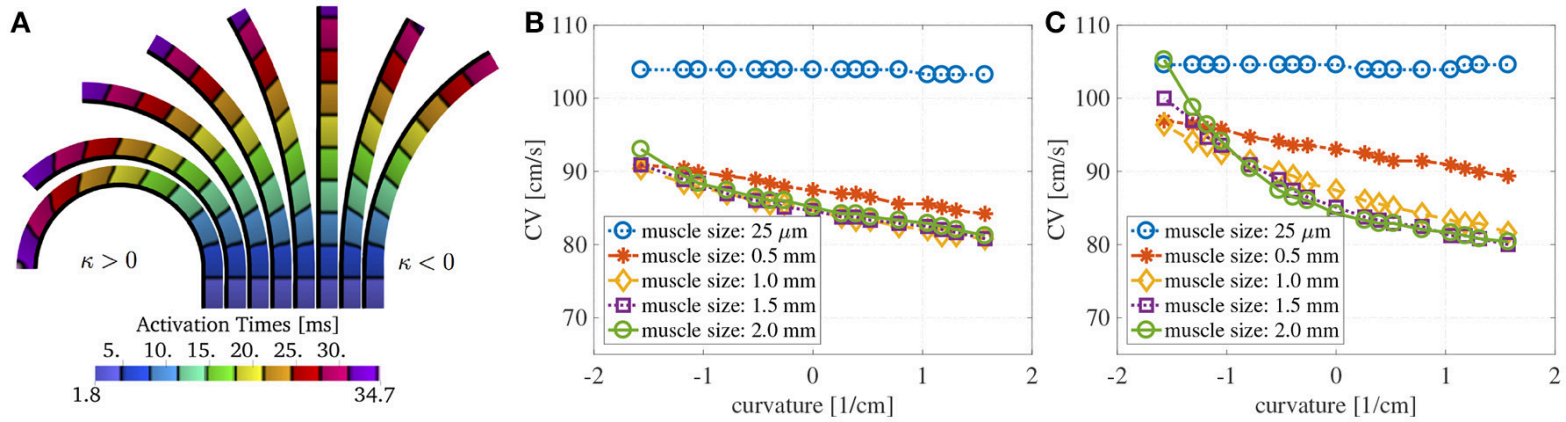
**(A)** Activation times at about 3.3 ms distance at different curvatures without bath-loading conditions for a muscle thickness of 1.5 mm. The change in shape of the wavefronts in the curved domains is clearly noticeable. The shapes depend on the sign and magnitude of the curvature κ. Note that the construction of the domain leads to different geometries for positive and negative curvatures. This is because that we keep the endocardial length fixed, but we allow the epicardial surface to become shorter or longer. **(B)** Endocardial CVs as a function of the curvature for several muscle thicknesses when an intracardiac bath of size ℓ_b_ = 6 mm is considered. **(C)** Endocardial CVs as a function of the curvature for several muscle thicknesses when intracardiac and extracardiac baths of size ℓ_b_ = 3 mm are considered. As for the case with no bath-loading conditions conduction endocardial CVs speed up for negative curvatures and slow down for positive curvatures **(B,C)**. The case of mucles thickness 25 μm correspends to the case of two-dimensional manifolds in three-dimensional simulations. When the muscle thickness ℓ_m_ is very small (25 μm), the CVs are independent of the curvature. In this case, the signal speed is strongly influenced by the bath conductivities. If ℓ_m_ > 1 mm, then muscle thickness does change much endocardial CVs but curvature does.

Finally, we show in Figure 5A the activation times at different curvatures for ℓ_m_ = 1.5 mm, using the parameters specified in Table 1. The black isolines are at distance 3.3 ms. The marked solid black line represents the endocardial surface where we measure the conduction velocities. As it can be seen, curvature greatly influences the activation times: the endocardial activation is slower for positive curvature (bending on the left) and faster for negative curvature (bending on the right) than in the straight case.

### 3.2. Endocardial CVs depends on muscle-thickness and curvature with bath-loading conditions

Here, we investigate the role of muscle thickness and curvature in presence of a bath. We consider a fixed intracardiac bath thickness ℓ_b_ = 6 mm and we test muscle thicknesses ℓ_m_ = 0.025, 0.5, 1, 1.5, and 2 mm. In Figure 5B, we show the endocardial CVs evaluated for the different muscle sizes. It can be noted here that in the case of muscle thickness ℓ_m_ = 25 μm, the conduction velocities are mainly dictated by the conductivity of the bath. A small thickness is sufficient to reveal the dependence on muscle curvature.

We also consider the case of intracardiac and extracardiac baths of thickness ℓ_b_ = 3 mm, testing again muscle thicknesses ℓ_m_ = 0.025, 0.5, 1, 1.5, and 2 mm. The corresponding CVs are shown in Figure 5C. Once again, if ℓ_m_ = 25 μm, the CVs are independent of the curvature. As expected, the extracardiac bath mainly influences endocardial CVs for muscle thickness smaller than 1 mm.

**Figure 6:**
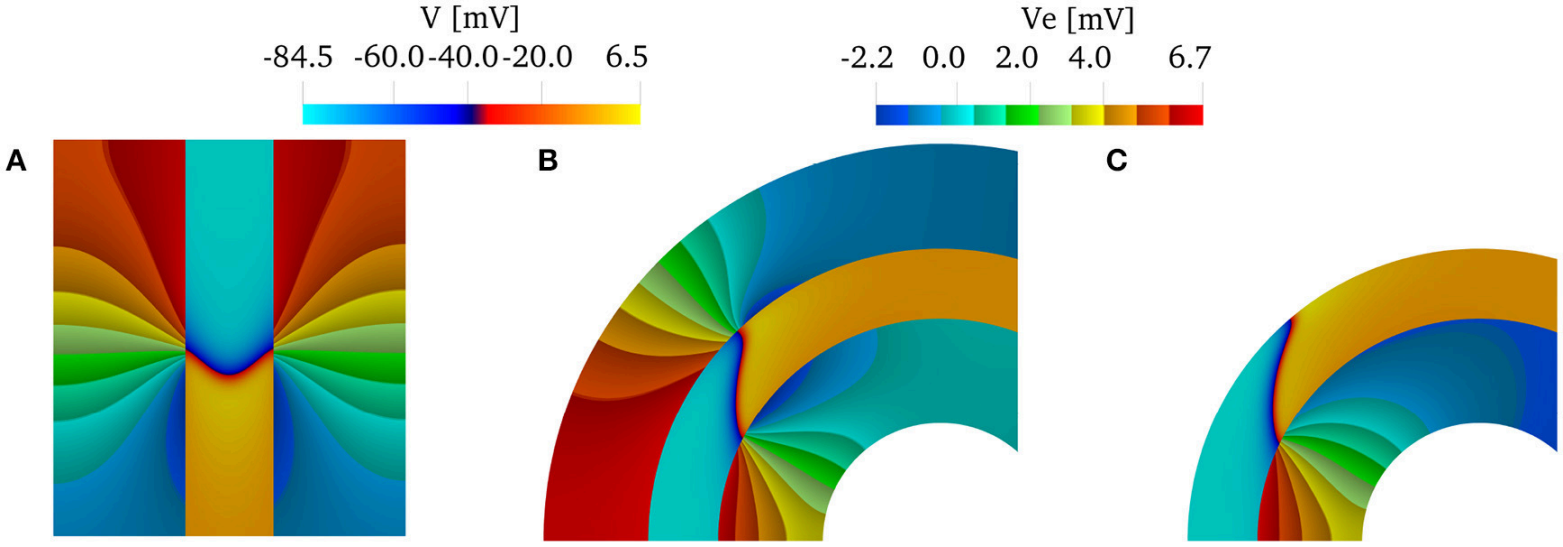
Transmembrane potentials in the muscle region and extracellular potentials in the bath regions at time *t* = 32 ms for a muscle thickness of 2 mm. **(A)** When the domain is straight (κ = 0 cm^−1^) and both intracardiac and extracardiac bath-loading conditions are considered, the wavefront takes the characteristic "V” shape. **(B)** When the domain is curved, the wavefront does not take the characteristic "V"-shape. **(C)** If we consider only the intracardiac bath, the endocardial CVs are still in good agreement with the case of intracardiac and extracardiac baths.)

We can conclude that if we are interested only in capturing endocardial CVs, using only an intracardiac bath is sufficient if the muscle thickness is greater than 1 mm. We show this in Figure 6, which shows the wavefront and the extracellular potential at time *t* = 32 ms for muscle thickness of 2 mm. As a reference, we show in Figure 6A the characteristic V-shaped wavefront when intracardiac and extracardiac bath are both considered in a straight domain. In the curved domain, Figure 6B, the front loses the characteristic V-shape. When only the intracardiac bath is considered, Figure 6C, the epicardial details of the front are lost, but the endocardial CVs are about the same. This can be noted by comparing the position of the endocardial fronts in Figures 6B,C.

**Figure 7:**
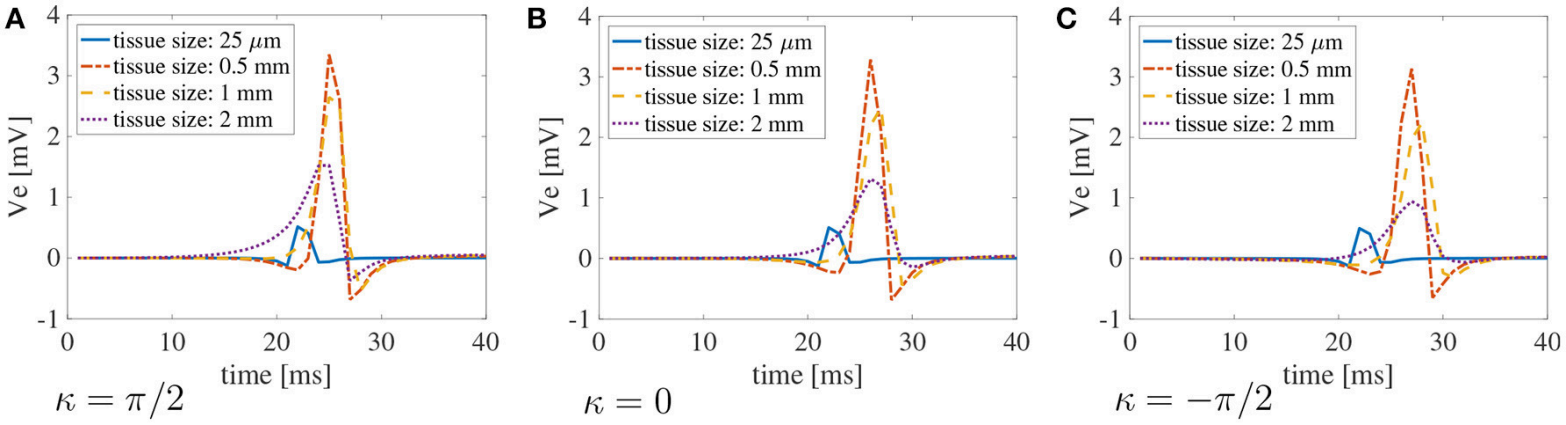
Bipolar signals ${V_{\text{e}}}^{2}-{V_{\text{e}}}^{1}$ recorded at 1 kHz for three selected curvatures κ = π/2 cm^−1^(A), 0 cm^−1^(B), andπ/2 cm^−1^**(C)** for bath size ℓ_b_ = 6 mm and varying muscle thickness ℓ_m_. The case of mucles thickness 25 μm correspends to the case of two-dimensional manifolds in three-dimensional simulations. When the muscle thickness is small (25 μm) the peak of the signal is shifted because the wavefront propagates faster. In that case, the amplitude of the signal is also extremely small. Substantial differences in the amplitude of the signals can be found at larger muscle thicknesses. The curvature of the domain does not play a major role in the recorded signals.

Finally, in Figure 7, we plot the bipolar signals measured on the endocardial surface, as explained in section 2.1, on three selected curvature: κ = π/2 cm^−1^, κ = 0 cm^−1^, and κ = −π/2 cm^−1^. Once again, if the muscle thickness is not accounted for, the peak of the signal is out of phase due to the increased CVs. Moreover, the amplitude of the signal is not accurate. Nonetheless, we can appreciate that the amplitude of the signals is greatly affected by the thickness of the muscle. No major differences in the signals can be noted for different curvatures.

### 3.3. Endocardial CVs depends on bath-size and curvature at fixed muscle thickness

In this test, we evaluate the size of the bath that is needed to correctly capture the endocardial CVs. Fixing the muscle thickness ℓ_m_ = 1.5 mm, we vary the bath thickness ℓ_b_.

**Figure 8:**
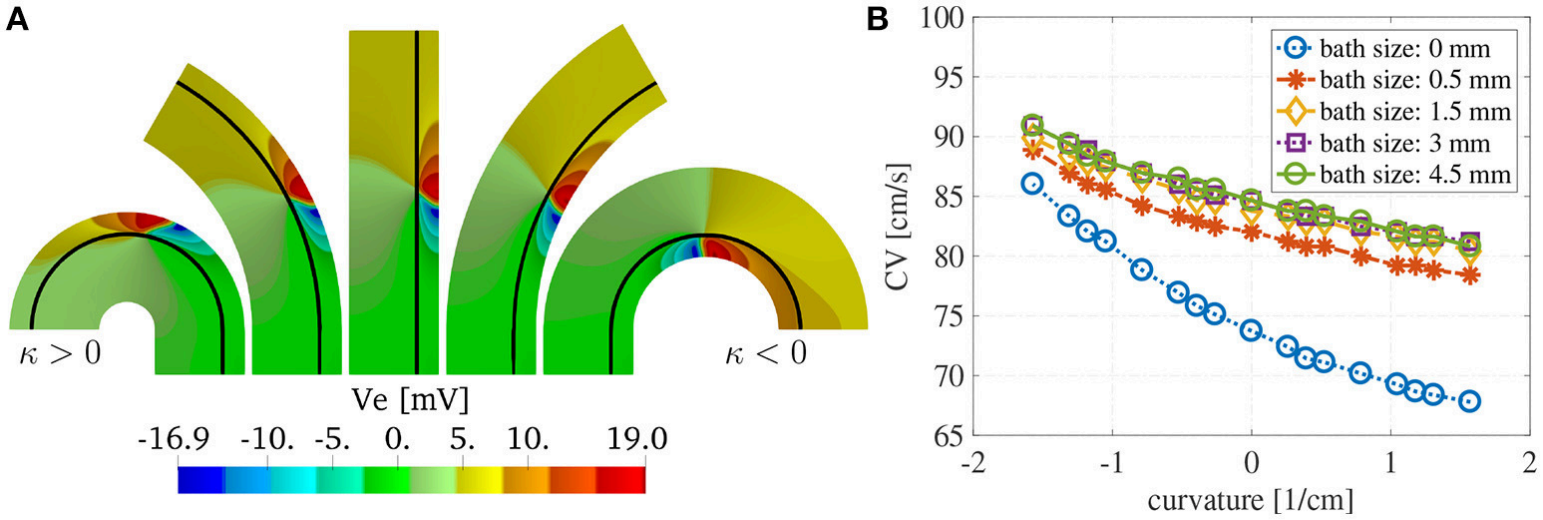
**(A)** Extracellular potential on selected curvatures for ℓ_m_ = 1.5 mm and ℓ_b_ = 6 mm at time *t* = 15 ms. The curvature of the domain changes the endocardial CVs as well as the shape of the wavefront. The wavefront is located at the sharp transition between negative (blue) and positive (red) voltages. **(B)** Endocardial CVs as a function of the curvature for muscle thickness ℓ_m_ = 1.5 mm and several thicknesses of the intracardiac bath. As expected the presence of bath-loading conditions increases the speed at which the wavefronts propagate. Positive (negative) curvature of the muscle decreases (increases) the endocardial CVs. A bath size of at least 1.5 mm was need to correctly capture the effects of the intracardiac bath-loading conditions.

We show in Figure 8A the extracellular potential *V*_e_ at *t* = 20 ms for some selected curvatures and ℓ_b_ = 6 mm. The solid black line corresponds to the endocardial interface. The front of the wave is localized in the muscle region where we have an abrupt change in the polarity of *V*_e_. We can appreciate from these plots the differences in the wavefront curvatures which depends both on the curvature of the domain and on the imposed boundary and interface conditions.

Figure 8B shows the dependence of the endocardial CVs on the curvature of the domain. We note here that for baths larger than 1.5 mm we measure the same CVs. This suggests that the bath should be at least of the size of the muscle to correctly capture the magnitude of the CVs.

**Figure 9:**
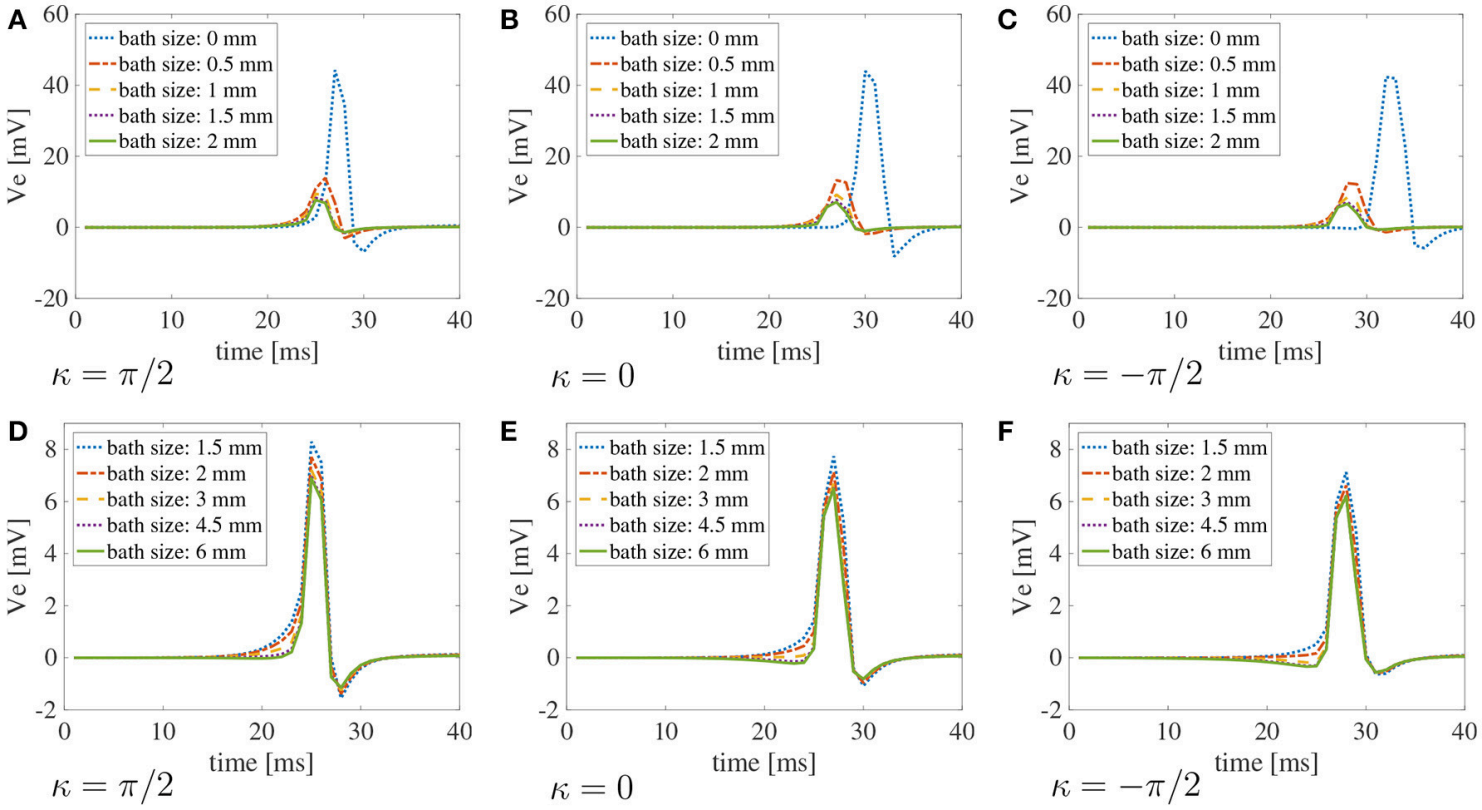
Bipolar signals ${V_{\text{e}}}^{2}-{V_{\text{e}}}^{1}$ recorded at 1 kHz for three selected curvatures κ = π/2 cm^−1^, 0 cm, π/2 cm^−1^ for different bath sizes and muscle thickness ℓ_m_ = 1.5 mm using the modified version of the Courtemanche atrial ionic model. **(A–C)** Bipolar signals for bath sizes between 0 and 2 mm. **(D–F)** Bipolar signals for bath sizes between 1.5 and 6 mm. An overlap of the data has been used between the top and bottom rows to better understand the differences in signals for different bath sizes. The curvature of the domain does not play a major role in the recorded signals. Large differences in the signal amplitudes can be found for bath sizes smaller than 2 mm. Although minor differences can also be noted for bath larger than 1.5 mm, the amplitude of the signals is well captured for baths of size at least 3 mm.

Similarly to the simplified case studied above, the conduction velocities strongly depend on the muscle curvature. Still, curvature has small effects on the bipolar signals. In Figure 9, we show the bipolar signals recorded at 1 kHz for the different bath sizes at three selected curvatures. Specifically, we show in Figures 9A–C the bipolar signals recorded for bath sizes between 0 and 2 mm, and in Figures 9D–F the bipolar signals recorded for bath sizes between 1.5 and 6 mm. These plots also suggest that a bath size of at least 1.5 mm is needed to correctly capture the bipolar signals.

### 3.4. Patient-specific left atrial posterior wall

**Figure 10:**
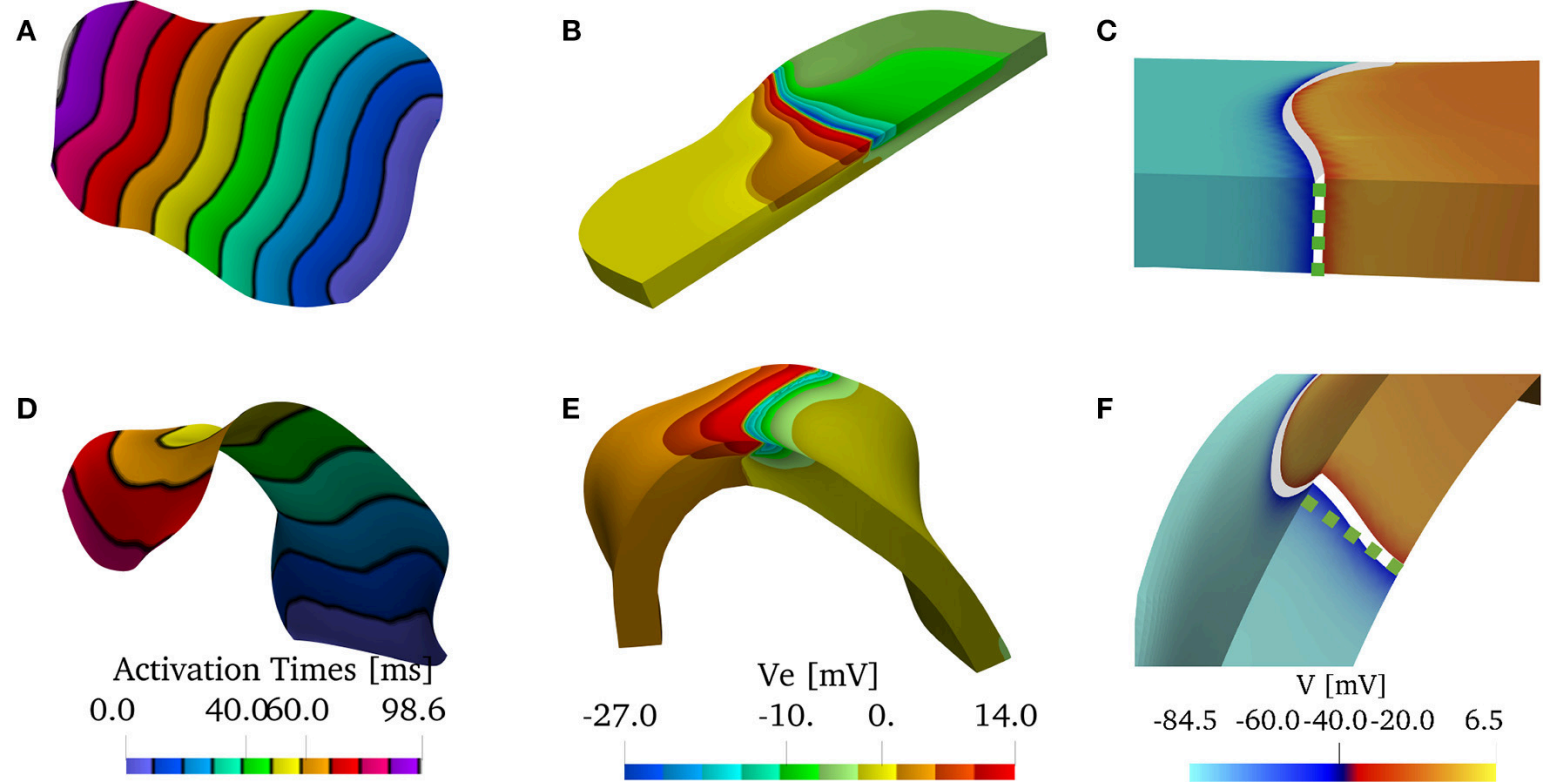
**(A–C)** Solution on the flattened posterior wall. **(D–F)** Solution on the patient-specific left atrial posterior wall. **(A,D)** Comparison of the endocardial activation times for a muscle size of 1.5 mm and the bath size of 2.85 mm. The curved geometry has slower conduction velocities. **(B,E)** Comparison of the extracellular potential *V*_e_ at *t* = 40 ms. **(C,F)** Comparison of the wavefront when no bath is considered. The front in the curved geometry deviates from being straight through the thickness of the muscle. We show in the dashed green line the straight profile.

In Figure 10, we show the solutions of the bidomain model on the patient-specific LAPW. More specifically, we show Figure 10A, the endocardial activation times (black isochrones at about 10 ms) when using the bath-loading conditions. In Figure 10B, we show the extracellular potential in the muscle and in the bath regions at time *t* = 40 ms. In Figure 10C, we show the shape of the wavefront at time *t* = 80 ms without a bath. The wavefront is highlighted in white, and the corresponding straight wavefront is depicted in the dashed green line. The corresponding results in the flattened LAPW are shown in Figures 10D–F. While in the flat geometry the wavefront remains straight, in the curved domain transversal conductivity and boundary conditions lead to a transmurally curved wavefront.

**Figure 11:**
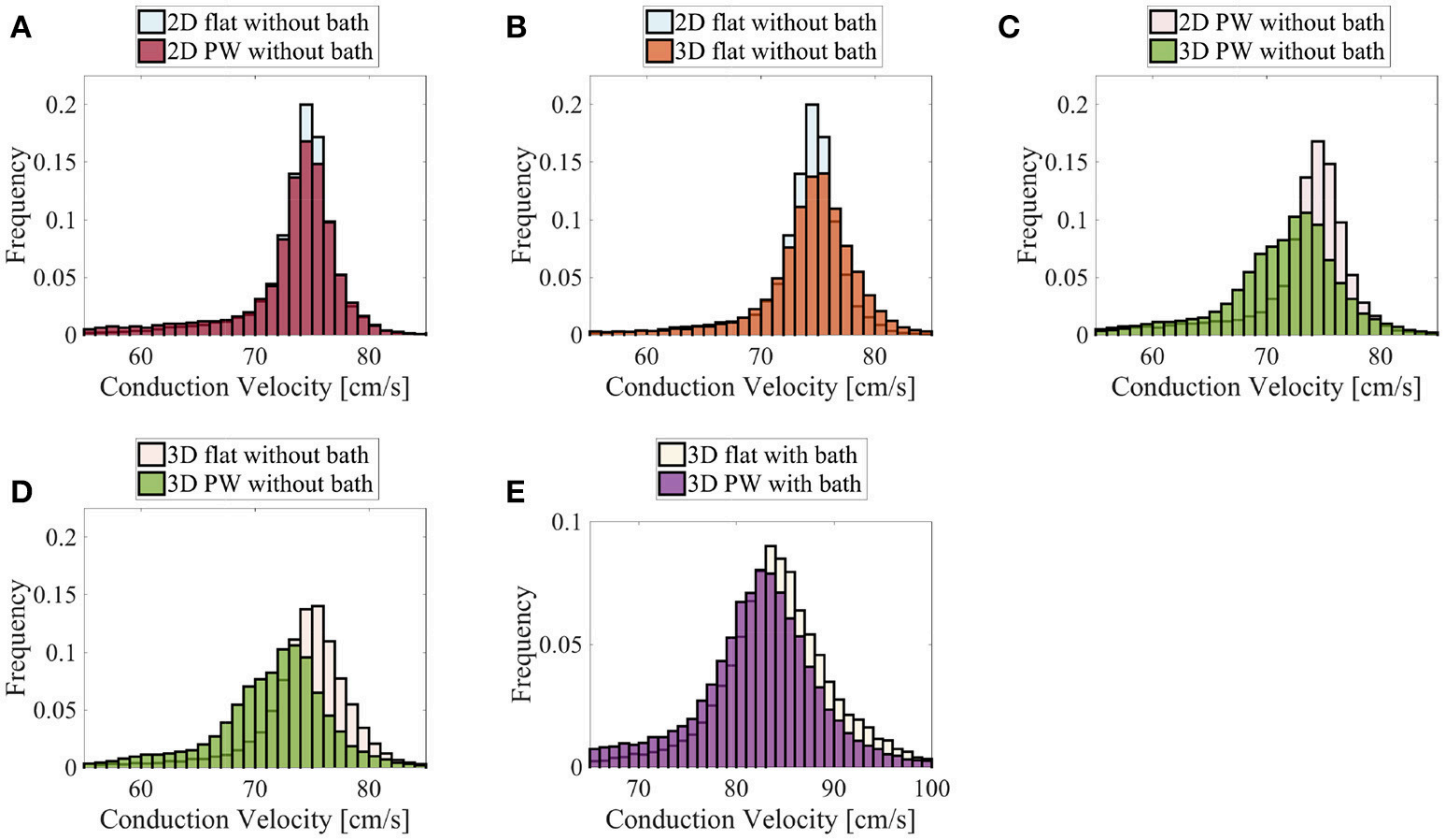
Distributions of the nodal endocardial CVs for the patient-specific LAPW. Simulations were run considering 2D (neglecting thickness) and 3D representations of the posterior wall (PW). To investigate the role of curvature, simulation were run on a flattened version of the posterior wall (flat). We also considered the case the of intracardiac bath-loading conditions (bath). The 2D simulations resulted in very similar distributions **(A)**, where the most frequent conduction velocities are around 74–76 cm/s. The conduction velocity distributions are also very similar when one considers a 2D and 3D flattened posterior wall **(B)**. On the contrary, in the actual curved posterior wall, the thickness of the tissue influences the distributions of the conduction velocities: the thickness of the muscle leads to a broader distribution of slower conduction velocities **(C)**. Similarly, the broader distribution of slower conduction velocities can be noted comparing the thick flattened and the actual posterior wall simulations **(D)**. When the bath is added to the simulation **(E)** the endocardial conduction velocities are substantially increased and the differences between the curved and the flattened geometries are reduced although they can still be appreciated.

Finally, we show in Figure 11 the distributions of the endocardial CV evaluated using (8). The CV of the LAPW and of the flattened LAPW have the same distribution if a bidimensional manifold is considered, where the most frequent conduction velocities are around 74–76 cm/s; see Figure 11A. Additionally, in the flattened LAPW, the thickness of the muscle does not influence the endocardial CV distribution; see Figure 11B. In the curved LAPW, the small thickness of the muscle is sufficient to slow down the endocardial conduction velocities; see Figure 11C. This is represented by the broader distribution of the 3D simulation in the CVs smaller than 70 cm/s. Additionally, the peak of the three-dimensional distribution corresponds to slightly slower CVs of about 72–75 cm/s. A similar difference can be noted also when comparing the distributions of the endocardial CVs for the flat and curved three-dimensional domains; see Figure 11D. When the bath-loading effects are considered; see Figure 11E, the differences are smaller but still noticeable: the peak of the distribution slows down from 85 to 84 cm/s and CVs smaller than 80 cm/s are more frequent throughout the domain.

## 4. Conclusions

Measurements of endocardial CVs can be used to characterize the electrophysiological health of the tissue substrate in patients with atrial fibrillation (AFib). CV is known to be affected by membrane excitability, front curvature, fiber orientation, and tissue anisotropy (Roberts et al., 1979; Rogers and McCulloch, 1994; Kléber and Rudy, 2004). In patients with persistent AFib, the morphological structure of the left atrium is correlated with pro-arrhythmic wave dynamics (Song et al., 2018). Heterogeneous atrial wall thickness is believed to contribute spiral wave localization or drift (Yamazaki et al., 2012; Biktasheva et al., 2015) and to support scroll waves underlying AFib maintenance (Yamazaki et al., 2012). The regional left atrial wall thickness has been strongly correlated with the dominant frequency, Shannon entropy, and the presence of complex fractionated atrial electrogram, associated with diseased tissue (Song et al., 2018). In addition, it has been shown that electrical dissociation between the epicardial layer and the endocardial layer during AFib increases stability and complexity of the AFib and is more pronounced in regions of thicker atrial wall (Eckstein et al., 2010). Along with wall thickness, curvature changes in wall geometry can also contribute to the initiation and maintenance of reentries by promoting wave-breaks (Rogers, 2002). Rogers showed that an expansion of the diffusive term of the monodomain model in terms of curvilinear coordinates reveals the role of curvature and muscle thickness on CVs (Rogers, 2002). For a spiral wave on a spherical manifold, an analytical expression for the angular velocities as a function of the curvature can be derived (Davydov and Zykov, 1991). These findings suggests that even under spatially uniform electrical and membrane properties, the complex geometry of the heart can destabilize wavefronts, causing fragmentation and complex activation patterns (Rogers, 2002). Rogers found that propagation was only affected by surface curvature when curvature was present in two directions (Rogers, 2002). In our simulations of an initially planar wave, we also found that that surface curvature in one direction does not influence propagation if the muscle is represented by a surface in three dimensions. Our simulations on a surface representation of a patient-specific left atrial posterior wall (LAPW) showed that the distributions of CVs are not influenced by the Gaussian curvature (curvature in two directions). As soon as muscle thickness is incorporated, curvature in one direction is sufficient to affect wavefront propagation speed.

We started our investigation by considering the role of thickness and curvature without bath-loading conditions. As expected, CVs are not influenced by muscle thickness if no curvature is imposed on the domain. Additionally, CVs are not influenced by curvature whenever the muscle thickness is negligible (e.g., 25 μm). This situation corresponds also to the manifold representation of the atria in some computational models (Vigmond et al., 2001; Zemlin et al., 2001; Virag et al., 2002; van Dam and van Oosterom, 2003; Weiser et al., 2010; Patelli et al., 2017). For larger muscle thicknesses, geometrical curvature influences the propagation of the electrical signal. For negative curvatures, the signal propagates faster, whereas for positive curvatures, the signal propagates with decreased CVs. For thin muscles (up to about 1 mm), the thicker the muscle the slower (faster) the CVs for positive (negative) curvatures. This relationship between curvature, muscle thickness, and CV is analogous to the well-known dependence of propagation efficacy on wavefront curvature (Tyson and Keener, 1988; Rogers and McCulloch, 1994; Rogers, 2002). We have shown that these changes in CV take place even without considering variations in the transmural properties of the muscle.

Under conditions with uniform transmural properties one might assume that a planar wavefront remains planar for any curvature. The term planar wavefront is used in analogy with the theory of plates, in which straight lines normal to the mid-surface remain normal to the mid-surface after deformation. In a similar sense, we understand that a planar wavefront is a front that is parallel to a straight line normal to the mid-surface and remains normal for any curvature of the domain. We have demonstrated in our tests that this assumption holds only under isotropic conditions. When anisotropy is introduced, the wavefront in curved domains does not remain planar. The transmural shape of the wavefront depends on two factors: (i) the anisotropy ratio and (ii) the boundary conditions. Even in more refined versions of the surface-based models of atrial electrophysiology (Chapelle et al., 2013), derived from asymptotic analysis averaging through the thickness, these factors are not well captured. For example, the surface-based models cannot represent the dissociation of endocardial and epicardial electrical activities during fibrillation. Single layer surface models have been improved by introducing a second layer to account for a more three-dimensional character of the fibrillatory conduction (Gharaviri et al., 2012; Labarthe et al., 2014; Coudière et al., 2017). Comparisons of these bilayer models with three-dimensional simulations are very limited and do not consider the possible influence of geometrical curvature on the electrical propagation. We have also shown that even under isotropic conditions where the fronts remain planar in curved domains, the endocardial CVs depend on the curvature. These results show that the fully three-dimensional atrial models are necessary to accurately capture the propagation of electrical signals and the corresponding conduction velocities on the endocardial surface.

A number of studies have shown that bath-loading conditions can increase conduction velocities (Roth, 1991, 1996; Henriquez et al., 1996; Srinivasan and Roth, 2004; Bishop et al., 2011). Comparing Figures 3 and 5, the CV for a muscle thickness of 25 μm increases from 74 to 104 cm/s in the presences of a bath. But as in the cases that omit the bath, curvature does not play a major role in determining the velocities. For muscle thicknesses between 0.5 and 2 mm, we have found that curvature in the presence of a bath acts to increase endocardial conduction velocities, but, in accordance with Roth (1991), the differences between the various thicknesses are smaller than they are without a bath. For positive curvatures, we have found that when no bath is considered, changes up 10% of the planar CVs can be measured. Although the curvature effect is smaller with bath-loading conditions, changes of up to 6% were found. These variations in CVs can actually be measured by electroanatomic mapping systems. We also found that changes in CVs for negative curvatures were more pronounced. For large negative curvature, we found variations of more than 10 cm/s. Even if these results may not be applied directly to the measurements of the CVs on the LAPW, which has mostly positive curvature, they highlight the strong correlation between structure and speed of propagation. We conclude that in the presence of bath-loading, three-dimensional atrial models are still necessary to accurately capture the propagation of electrical signals and their conduction velocities. To reduce the computational cost when bath-loading conditions are considered, and the main interest is the evaluation of endocardial conduction velocity during normal propagation, it could be possible to consider a uniform atrial thickness of about 1 mm. On the other hand, this approximation may fail to correctly represent endo-epicardial dissociation and transmural breakthrough during Afib. In accordance to the results shown by Bishop and Plank (2011), in our simplified test case, fixing the muscle thickness at 1.5 mm, a bath size larger than 1.5 mm was necessary to correctly capture endocardial CVs. The effects of curvature on CVs are important for all bath sizes and the same considerations as in the case with no bath-loading conditions described above hold.

The above considerations, drawn from a simple two-dimensional test case, were found to also hold in realistic geometries. Specifically, we have reconstructed a human model of the LAPW, assuming a uniform fiber field, in which the direction of anisotropy was obtained from a scalar harmonic potential. We solved the anisotropic bidomain model considering: (i) only the endocardial surface; (ii) only the atrial muscle with thickness 1.5 mm; and (iii) the atrial muscle with an intracardiac bath of 2.85 mm of thickness. Additionally, in patient-specific geometries, it is not possible to precisely control the direction of propagation. Therefore, to study the role of curvature, we recreated a flattened version of the LAPW. Endocardial conduction velocities were computed at each vertex of the triangulation of the domain using weighted averages based on the gradients of the activation times. Comparing the distributions in the various scenarios, we have concluded that curvature and muscle thickness can strongly influence the measured conduction velocities. In fact, we have found a shift in the peak CVs, with a reduction of about 2–4 cm/s, when comparing the distributions of the three-dimensional patient-spceific geometry with those of a manifold or a flattened representation of the LAPW. More importantly, when muscle thickness and curvature are included, the overall distributions have slower decays on the left and faster decays on the right. A two-sample *t*-test (Snedecor and William, 1989) has determined that the difference in two distributions means is statistically significant (*p* value = 0). This behavior, shown in Figure 11, leads to an overall slow down of the propagation of the electrical signal.

Because clinical CV maps are derived from extracellular electrograms, we also investigated how bipolar signals are affected by muscle thickness, curvature, and bath size. To mimic clinical conditions, the unipolar signals were sampled at 1 kHz at two points on the endocardial surface at a distance of 2 mm. We found that curvature does not play any substantial role on the electrogram morphology. On the other hand, muscle thickness and bath size can influence the amplitude of the signals. Still, the differences for a bath size larger than 1.5 mm were small. A major difference was found when approximating the muscle thickness with a bidimensional manifold. This corresponds to the test with a muscle thickness of 25 μm. In this case, the amplitude and the shape of the signals were very different from the cases in which the muscle thickness was between 0.5 and 2 mm. In particular, we recorded a maximum peak smaller than 1 mV for 25 μm muscle thickness, while for thicker muscles the peak was greater than 1 mV. Given the faster CVs for the thin muscle case, the maximum peak was recorded earlier than for thicker muscles. We also note that, accordingly to the discussion above on CVs, the time at which the peak bipolar signal is recorded depends on the muscle curvature. This finding suggests the use of three-dimensional models for atrial electrophysiology for accurately simulating surface electrogarms.

To verify that our findings were not largely affected by the numerical methods used, we also solved the bidomain model with a cubic reaction term in place of the Courtemanche ionic model. This simple reaction model can be used to represent a propagating front guaranteeing a second order convergence of the numerical method used herein (Rossi and Griffith, 2017). The details of this model and the results can be found in the Supplementary Material. Except for some differences in the details of the registered bipolar signals due to the different shape of the propagating pulse, the same qualitative behavior with respect to muscle thickness, curvature, and bath size was found. This suggests that our findings obtained using numerical methods with a suboptimal order of convergence are correct.

In conclusion, we have found evidence that even under homogeneous conditions, a surface-based model of the atria is not accurate in capturing the endocardial CVs and magnitude of the endocardial bipolar signals. In general, the change in CV for different curvatures is a function of muscle thickness (Figure 3). This effect is reduced in the presence of an adjoining bath. For the left atrial posterior wall with positive curvature, the electrical signal propagates more slowly on the endocardial surface than it would on a flat region. It has been shown in the ventricles that regions of slow conduction regions are correlated with anatomical sites critical for tachycardia (Irie et al., 2015). This slowing seen during curvature may be exacerbated under compromised electrophysiological conditions. The effects of geometry and bath-loading on conduction is important if CV is to be used as an index to indicate regions with fibrosis or poor conductivity. From the computational point of view, the findings suggest that models of atrial electrophysiology used to guide and understand endocardial catheter measurements should be fully three-dimensional and account for bath-loading effects with a simulated bath size of at least 1.5 mm was necessary for our simulation to get consistent CV measurements.

## 5. Limitations

Our simulations had several limitations. First, we considered uniform muscle thicknesses between 0.5 mm and 2 mm and uniform curvatures. The atrial wall (Bishop et al., 2015) thickness varies and has been shown to affect wavefront dynamics in atrial fibrillation (Rogers, 2002; Biktasheva et al., 2015; Song et al., 2018). Although the cases we considered are within the range of left atrial wall thicknesses (Bishop et al., 2015), measurements of the LAPW have shown that the muscle thickness can be as large as 5,mm superiorly and 8 mm inferiorly (Platonov et al., 2008). Additionally, the average the atrial wall thickness is about 2.73 mm (Pashakhanloo et al., 2016). We have shown that the thicker the muscle, the more important is to consider a three-dimensional model of cardiac electrophysiology, but the endocardial CVs are captured with good accuracy even when loading-bath conditions are considered and a thickness of about 1.5 mm is used. Introducing non-uniform wall thickness in the patient-specific simulations can be challenging because a description of the epicardial surface is not readily available by endocardial electroanatomical maps or easily discernable from standard imaging. Finally, we have mainly considered propagation along a strand of tissue without considering transmural propagation. In fact, we have shown that in an anisotropic domain with no bath, a planar wavefront becomes curved if the domain has curvature. A more detailed study should be carried out to have proper insights on transmural propagation.

## Data availability statement

BeatIt, the C++ code created for this study is publicly available on GitHub at the address github.com/rossisimone/beatit.git.

## Author contributions

SR: conception, design, code implementation, drafting, data preprocessing, data analysis, and interpretation of data; SG: Conception, design, Patient-specific data acquisition, critical revision; BG and CH: Design, critical revision.

### Conflict of interest statement

The authors declare that the research was conducted in the absence of any commercial or financial relationships that could be construed as a potential conflict of interest.
