# Supplementary material for: Muscle Thickness and Curvature Influence Atrial Conduction Velocities
Source: Front Physiol. 2018 Oct 29;9:1344. doi: 10.3389/fphys.2018.01344 (PMC6215968; doi:10.3389/fphys.2018.01344)
Supplement: Supplementary file 1 [file Presentation_1.pdf]

Supplementary Material:
Muscle Thickness and Curvature Influence Atrial Conduction Velocities

1 MACROSCOPIC DERIVATION OF THE BIDOMAIN MODEL

We assume that the homogenization assumptions hold, and we derive the bidomain model phenomenologically, starting from charge conservation in quasi-static conditions. Consider the intracellular and extracellular fluxes,

$$\boldsymbol{j}_{\mathrm{i}} = -\boldsymbol{D}_{\mathrm{i}}\nabla V_{\mathrm{i}}, \quad \boldsymbol{j}_{\mathrm{e}} = -\boldsymbol{D}_{\mathrm{e}}\nabla V_{\mathrm{e}}, \tag{S1}$$

in which $\boldsymbol{D}_{\mathrm{i}} = \sigma_{\mathrm{i}}^{\mathrm{t}}\boldsymbol{I} + \left(\sigma_{\mathrm{i}}^{\mathrm{f}} - \sigma_{\mathrm{i}}^{\mathrm{t}}\right)\mathbf{f}\otimes\mathbf{f}$ and $\boldsymbol{D}_{\mathrm{e}} = \sigma_{\mathrm{e}}^{\mathrm{t}}\boldsymbol{I} + \left(\sigma_{\mathrm{e}}^{\mathrm{f}} - \sigma_{\mathrm{e}}^{\mathrm{t}}\right)\mathbf{f}\otimes\mathbf{f}$ are the intracellular and extracellular conductivity tensors, and V_{i} and V_{e} are the intracellular and extracellular electric potentials. Here, $\sigma_{\mathrm{i}}^{\mathrm{f}}$ and $\sigma_{\mathrm{i}}^{\mathrm{t}}$ denote the tissue conductivities along and across the fiber direction in the intracellular space, and $\sigma_{\mathrm{e}}^{\mathrm{f}}$ and $\sigma_{\mathrm{e}}^{\mathrm{t}}$ denote the extracellular conductivities, in which $\boldsymbol{I}$ is the identity matrix. Denoting with $I_{\mathrm{i}}^{\mathrm{v}}$, $I_{\mathrm{e}}^{\mathrm{v}}$ and I_{m} the volumetric intracellular extracellular and membrane current densities, charge conservation can be written as

$$-\nabla\cdot\boldsymbol{j}_{\mathrm{i}} = -I_{\mathrm{m}} + I_{\mathrm{i}}^{\mathrm{v}}, \quad -\nabla\cdot\boldsymbol{j}_{\mathrm{e}} = I_{\mathrm{m}} + I_{\mathrm{e}}^{\mathrm{v}}, \tag{S2}$$

Taking the divergence of (S1), we find

$$-\nabla\cdot(\boldsymbol{D}_{\mathrm{i}}\nabla V_{\mathrm{i}}) = \nabla\cdot\boldsymbol{j}_{\mathrm{i}}, \tag{S3a}$$
$$-\nabla\cdot(\boldsymbol{D}_{\mathrm{e}}\nabla V_{\mathrm{e}}) = \nabla\cdot\boldsymbol{j}_{\mathrm{e}}. \tag{S3b}$$

Substituting the relations from charge conservation(S2),

$$-\nabla\cdot(\boldsymbol{D}_{\mathrm{i}}\nabla V_{\mathrm{i}}) = -I_{\mathrm{m}} + I_{\mathrm{i}}^{\mathrm{v}}, \tag{S4a}$$
$$-\nabla\cdot(\boldsymbol{D}_{\mathrm{e}}\nabla V_{\mathrm{e}}) = I_{\mathrm{m}} + I_{\mathrm{e}}^{\mathrm{v}}. \tag{S4b}$$

The transmembrane current has two components: a capacitive current due to the ability of the cellular membrane to separate charged ions, and a resistive current due to the ionic channels. Introducing the membrane capacitance C_{m}, the cellular surface-to-volume ratio χ, and the transmembrane potential $V = V_{\mathrm{i}} - V_{\mathrm{e}}$, the membrane current can be written as

$$I_{\mathrm{m}} = \chi\left(C_{\mathrm{m}}\partial_t V + I_{\mathrm{ion}}\right). \tag{S5}$$

Using the above definition (S5) in (S4a) and (S4b), we obtain the parabolic-parabolic bidomain model

$$\nabla\cdot(\boldsymbol{D}_{\mathrm{i}}\nabla V_{\mathrm{i}}) = \chi\left(C_{\mathrm{m}}\partial_t V + I_{\mathrm{ion}}\right) - I_{\mathrm{i}}^{\mathrm{v}}, \tag{S6a}$$
$$\nabla\cdot(\boldsymbol{D}_{\mathrm{e}}\nabla V_{\mathrm{e}}) = -\chi\left(C_{\mathrm{m}}\partial_t V + I_{\mathrm{ion}}\right) - I_{\mathrm{e}}^{\mathrm{v}}. \tag{S6b}$$

Summing (S6a) and (S6b) and using $V_\text{i} = V + V_\text{e}$, we find the parabolic-elliptic bidomain model

$$\nabla \cdot (\boldsymbol{D}_\text{i} \nabla V) + \nabla \cdot (\boldsymbol{D}_\text{i} \nabla V_\text{e}) = \quad \chi \left(C_\text{m} \partial_t V + I_\text{ion}\right) - I_\text{i}^\text{v}, \tag{S7a}$$

$$\nabla \cdot (\boldsymbol{D}_\text{i} \nabla V) + \nabla \cdot \left(\left(\boldsymbol{D}_\text{i} + \boldsymbol{D}_\text{e}\right) \nabla V_\text{e}\right) = -I_\text{total}^\text{v}. \tag{S7b}$$

An important indicator in cardiac electrophysiology is the rate of change of the transmembrane potential. We introduce it as a separate variable $Q = \partial_t V$ (Rossi and Griffith, 2017a), such that

$$\partial_t V = Q \tag{S8a}$$

$$0 = \nabla \cdot (\boldsymbol{D}_\text{i} \nabla V) + \nabla \cdot (\boldsymbol{D}_\text{i} \nabla V_\text{e}) - \chi \left(C_\text{m} Q + I_\text{ion}\left(V, \boldsymbol{w}, \boldsymbol{c}\right)\right) + I_\text{i}^\text{v}, \tag{S8b}$$

$$0 = \nabla \cdot (\boldsymbol{D}_\text{i} \nabla V) + \nabla \cdot \left(\left(\boldsymbol{D}_\text{i} + \boldsymbol{D}_\text{e}\right) \nabla V_\text{e}\right) - I_\text{total}^\text{v}. \tag{S8c}$$

The bidomain model is supplemented with a model for the ionic currents I_ion given by a set of ODEs

$$\partial_t \boldsymbol{w} = \boldsymbol{g}\left(V, \boldsymbol{w}\right). \tag{S9}$$

Here the vector $\boldsymbol{w}$ contains the gating variables, a set of internal variables describing the current state of the ionic channels. The ionic model (S9) describes the dynamics of the channel opening and closing as a function of the transmembrane potential V.

We refer to (Henriquez et al., 1996; Keener and Sneyd, 1998; Griffith and Peskin, 2013; Franzone et al., 2014) for more details on models of cardiac electrophysiology.

2 THE PARABOLIC-ELLIPTIC BIDOMAIN MODEL

Denote with $\Omega \subset \mathbb{R}^3$ a Lipschitz bounded domain and $\Gamma = \partial\Omega$ its boundary with corresponding unit normal vector $\mathbf{n}$. The muscle domain Ω_m is in contact with a conducting medium Ω_b, representing either the intracavitary blood or an extracardiac bath, such that $\Omega_\text{m} \cup \Omega_\text{b} = \Omega$. The sets Γ_m and Γ_b represent the boundary of the muscle and bath domains. The interface between the muscle and the bath domain is denoted with Γ_i. The bidomain model with bath-loading conditions reads

$$\partial_t V = Q, \qquad \text{in } \Omega_\text{m} \times]0, T[, \tag{S10a}$$

$$0 = \nabla \cdot (\boldsymbol{D}_\text{i} \nabla V) + \nabla \cdot (\boldsymbol{D}_\text{i} \nabla V_\text{e}) - \chi \left(C_\text{m} Q + I_\text{ion}\right) + I_\text{i}^\text{v}, \qquad \text{in } \Omega_\text{m} \times]0, T[, \tag{S10b}$$

$$0 = \nabla \cdot (\boldsymbol{D}_\text{i} \nabla V) + \nabla \cdot \left(\left(\boldsymbol{D}_\text{i} + \boldsymbol{D}_\text{e}\right) \nabla V_\text{e}\right) - I_\text{total}^\text{v}, \qquad \text{in } \Omega_\text{m} \times]0, T[, \tag{S10c}$$

$$\nabla \cdot (\boldsymbol{D}_\text{b} \nabla V_\text{b}) = I_\text{b}, \qquad \text{in } \Omega_\text{b} \times]0, T[, \tag{S10d}$$

together with the ionic model

$$\partial_t \boldsymbol{w} = \boldsymbol{g}\left(V, \boldsymbol{w}\right), \qquad \text{in } \Omega_\text{m} \times]0, T[, \tag{S11a}$$

where we set the initial conditions

$$V\left(\boldsymbol{x}, 0\right) = V_0\left(\boldsymbol{x}\right), \qquad \text{in } \Omega_\text{m}, \tag{S12a}$$

$$Q\left(\boldsymbol{x}, 0\right) = Q_0\left(\boldsymbol{x}\right), \qquad \text{in } \Omega_\text{m}, \tag{S12b}$$

$$\boldsymbol{w}\left(\boldsymbol{x}, 0\right) = \boldsymbol{w}\left(\boldsymbol{x}\right), \qquad \text{in } \Omega_\text{m}, \tag{S12c}$$

and the boundary conditions

$$\mathbf{n}\cdot\left(\boldsymbol{D}_{\mathrm{i}}\nabla V_{\mathrm{i}}\right)=\mathbf{n}\cdot\left(\boldsymbol{D}_{\mathrm{i}}\nabla\left(V+V_{\mathrm{e}}\right)\right)=I_{\mathrm{i}}^{\mathrm{s}}, \qquad \text{on } \Gamma_{\mathrm{m}}\times\left]0,T\right[, \tag{S13a}$$

$$\mathbf{n}\cdot\left(\boldsymbol{D}_{\mathrm{e}}\nabla V_{\mathrm{e}}\right)=I_{\mathrm{e}}^{\mathrm{s}}, \qquad \text{on } \Gamma_{\mathrm{m}}\times\left]0,T\right[, \tag{S13b}$$

$$\mathbf{n}\cdot\left(\boldsymbol{D}_{\mathrm{b}}\nabla V_{\mathrm{b}}\right)=0, \qquad \text{on } \Gamma_{\mathrm{b}}\times\left]0,T\right[, \tag{S13c}$$

with $\mathbf{n}$ being the normal to the surface. Along Γ_{i} we impose the interface conditions

$$V_{\mathrm{b}}=V_{\mathrm{e}}, \qquad \text{on } \Gamma_{\mathrm{i}}\times\left]0,T\right[, \tag{S14a}$$

$$\mathbf{n}_{\mathrm{i}}\cdot\left(\boldsymbol{D}_{\mathrm{b}}\nabla V_{\mathrm{b}}\right)=-\mathbf{n}_{\mathrm{i}}\cdot\left(\boldsymbol{D}_{\mathrm{e}}\nabla V_{\mathrm{e}}\right). \qquad \text{on } \Gamma_{\mathrm{i}}\times\left]0,T\right[, \tag{S14b}$$

In (S14b) $\mathbf{n}_{\mathrm{i}}=\mathbf{n}_{\mathrm{b}}=-\mathbf{n}_{\mathrm{e}}$ is the normal to the muscle-bath interface Γ_{i}.

3 SPACE DISCRETIZATION

A detailed description of discretization and solver for the bidomain equation is given in Vigmond et al. (2008). We present here a common finite element discretizationPlank et al. (2005); Franzone et al. (2006); Seemann et al. (2006); Pathmanathan et al. (2010); Bishop and Plank (2011); Landajuela et al. (2018). The derivation below follows the one presented by Bendahmane and Chamakuri (2017).

Denote by

$$(u,v)_{\Omega}=\int_{\Omega}uv,$$

the $L_2(\Omega)$ inner-product and by

$$\langle u,v\rangle_{\Gamma}=\int_{\Gamma}uv$$

a functional on a boundary. The weak problem reads: for every $t\in[0,T]$, find $V^h\in\mathcal{V}_{\mathrm{m}}^h$,$V_{\mathrm{e}}^h\in\mathcal{V}_{\mathrm{m}}^h$ and $V_{\mathrm{b}}^h\in\mathcal{V}_{\mathrm{b}}^h$ such that

$$\left(\partial_t V^h,\varphi^h\right)_{\Omega_{\mathrm{m}}}=\left(Q^h,\varphi^h\right)_{\Omega_{\mathrm{m}}}, \tag{S15a}$$

$$\begin{aligned}0=&-\left(\boldsymbol{D}_{\mathrm{i}}\nabla V^h,\nabla\phi^h\right)_{\Omega_{\mathrm{m}}}-\left(\boldsymbol{D}_{\mathrm{i}}\nabla V_{\mathrm{e}}^h,\nabla\phi^h\right)_{\Omega_{\mathrm{m}}}\\&-\left(\chi C_{\mathrm{m}}Q^h,\phi^h\right)_{\Omega_{\mathrm{m}}}-\left(\chi I_{\mathrm{ion}}^h,\phi^h\right)_{\Omega_{\mathrm{m}}}+\left\langle I_{\mathrm{i}}^{\mathrm{s}},\phi^h\right\rangle_{\Gamma_{\mathrm{m}}},\end{aligned} \tag{S15b}$$

$$\begin{aligned}0=&-\left(\boldsymbol{D}_{\mathrm{i}}\nabla V^h,\nabla\xi^h\right)_{\Omega_{\mathrm{m}}}-\left(\left(\boldsymbol{D}_{\mathrm{i}}+\boldsymbol{D}_{\mathrm{e}}\right)\nabla V_{\mathrm{e}}^h,\nabla\xi^h\right)_{\Omega_{\mathrm{m}}}\\&-\left(I_{\mathrm{total}}^{\mathrm{v}},\xi^h\right)_{\Omega_{\mathrm{m}}}+\left\langle\boldsymbol{D}_{\mathrm{e}}\nabla V_{\mathrm{e}}^h\cdot\mathbf{n}_i,\xi^h\right\rangle_{\Gamma_{\mathrm{i}}}+\left\langle I_{\mathrm{i}}^{\mathrm{s}}+I_{\mathrm{e}}^{\mathrm{s}},\phi^h\right\rangle_{\Gamma_{\mathrm{m}}},\end{aligned} \tag{S15c}$$

$$0=-\left(\boldsymbol{D}_{\mathrm{b}}\nabla V_{\mathrm{b}}^h,\nabla\eta^h\right)_{\Omega_{\mathrm{b}}}+\left\langle\boldsymbol{D}_{\mathrm{e}}\nabla V_{\mathrm{e}}^h\cdot\mathbf{n}_i,\xi^h\right\rangle_{\Gamma_{\mathrm{i}}}+\left(I_{\mathrm{b}},\eta^h\right)_{\Omega_{\mathrm{b}}}, \tag{S15d}$$

for all $\varphi^h\in\mathcal{V}_{\mathrm{m}}^h$, $\xi^h\in\mathcal{V}_{\mathrm{m}}^h$, and $\eta^h\in\mathcal{V}_{\mathrm{b}}^h$. In a monolithic formulation of the problem, the continuity of the potential at the interface, that is the interface conditions (S14a), can be enforced by simply imposing $\xi^h=\eta^h$ on Γ_{i}. Continuity of the fluxes on the muscle-bath interface Γ_{i}, represented by the interface

condition (S14b), follows directly from summing (S15c) and (S15d). In this way we obtain the problem: for every $t \in [0, T]$, find $V^h \in \mathcal{V}_{\mathrm{m}}^h$,$V_{\mathrm{e}}^h \in \mathcal{V}_{\mathrm{m}}^h$ and $V_{\mathrm{b}}^h \in \mathcal{V}_{\mathrm{b}}^h$ such that

$$\left(\partial_t V^h, \varphi^h\right)_{\Omega_{\mathrm{m}}} = \left(Q^h, \varphi^h\right)_{\Omega_{\mathrm{m}}}, \tag{S16a}$$

$$\begin{aligned} 0 = &- \left(\boldsymbol{D}_{\mathrm{i}} \nabla V^h, \nabla \phi^h\right)_{\Omega_{\mathrm{m}}} - \left(\boldsymbol{D}_{\mathrm{i}} \nabla V_{\mathrm{e}}^h, \nabla \phi^h\right)_{\Omega_{\mathrm{m}}} - \left(\chi C_{\mathrm{m}} Q^h, \phi^h\right)_{\Omega_{\mathrm{m}}} \\ &- \left(\chi I_{\mathrm{ion}}^h, \phi^h\right)_{\Omega_{\mathrm{m}}} + \left\langle I_{\mathrm{i}}^{\mathrm{s}}, \phi^h\right\rangle_{\Gamma_{\mathrm{m}}}, \end{aligned} \tag{S16b}$$

$$\begin{aligned} 0 = &- \left(\boldsymbol{D}_{\mathrm{i}} \nabla V^h, \nabla \xi^h\right)_{\Omega_{\mathrm{m}}} - \left(\left(\boldsymbol{D}_{\mathrm{i}} + \boldsymbol{D}_{\mathrm{e}}\right) \nabla V_{\mathrm{e}}^h, \nabla \xi^h\right)_{\Omega_{\mathrm{m}}} - \left(\boldsymbol{D}_{\mathrm{b}} \nabla V_{\mathrm{b}}^h, \nabla \eta^h\right)_{\Omega_{\mathrm{b}}} \\ &- \left(I_{\mathrm{total}}^{\mathrm{v}}, \xi^h\right)_{\Omega_{\mathrm{m}}} + \left(I_{\mathrm{b}}, \eta^h\right)_{\Omega_{\mathrm{b}}} + \left\langle I_{\mathrm{i}}^{\mathrm{s}} + I_{\mathrm{e}}^{\mathrm{s}}, \phi^h\right\rangle_{\Gamma_{\mathrm{m}}}, \end{aligned} \tag{S16c}$$

for all $\varphi^h, \phi^h, \xi^h \in \mathcal{V}_{\mathrm{m}}^h$, and for all $\eta^h \in \mathcal{V}_{\mathrm{b}}^h$ such that $\xi^h = \eta^h$ on Γ_{i}. Defining

$$U^h = \begin{cases} V_{\mathrm{e}}^h & \text{if } \boldsymbol{x} \in \Omega_{\mathrm{m}} \\ V_{\mathrm{b}}^h & \text{if } \boldsymbol{x} \in \Omega_{\mathrm{b}} \end{cases}, \quad \zeta^h = \begin{cases} \xi^h & \text{if } \boldsymbol{x} \in \Omega_{\mathrm{m}} \\ \eta^h & \text{if } \boldsymbol{x} \in \Omega_{\mathrm{b}} \end{cases}, \tag{S17}$$

and

$$\boldsymbol{D} = \begin{cases} \boldsymbol{D}_{\mathrm{i}} + \boldsymbol{D}_{\mathrm{e}} & \text{if } \boldsymbol{x} \in \Omega_{\mathrm{m}} \\ \boldsymbol{D}_{\mathrm{b}} & \text{if } \boldsymbol{x} \in \Omega_{\mathrm{b}} \end{cases}, \quad I^{\mathrm{v}} = \begin{cases} -I_{\mathrm{total}}^{\mathrm{v}} & \text{if } \boldsymbol{x} \in \Omega_{\mathrm{m}} \\ I_{\mathrm{b}} & \text{if } \boldsymbol{x} \in \Omega_{\mathrm{b}} \end{cases} \tag{S18}$$

we can write the weak form as: for every $t \in [0, T]$, find $V^h, Q^h \in \mathcal{V}_{\mathrm{m}}^h$ and $U^h \in \mathcal{S}^h$

$$\left(\partial_t V^h, \varphi^h\right)_{\Omega_{\mathrm{m}}} = \left(Q^h, \varphi^h\right)_{\Omega_{\mathrm{m}}}, \tag{S19a}$$

$$\begin{aligned} 0 = &- \left(\boldsymbol{D}_{\mathrm{i}} \nabla V^h, \nabla \phi^h\right)_{\Omega_{\mathrm{m}}} - \left(\boldsymbol{D}_{\mathrm{i}} \nabla U^h, \nabla \phi^h\right)_{\Omega_{\mathrm{m}}} - \left(\chi C_{\mathrm{m}} Q^h, \phi^h\right)_{\Omega_{\mathrm{m}}} \\ &- \left(\chi I_{\mathrm{ion}}^h, \phi^h\right)_{\Omega_{\mathrm{m}}} + \left\langle I_{\mathrm{i}}^{\mathrm{s}}, \phi^h\right\rangle_{\Gamma_{\mathrm{m}}}, \end{aligned} \tag{S19b}$$

$$0 = - \left(\boldsymbol{D}_{\mathrm{i}} \nabla V^h, \nabla \zeta^h\right)_{\Omega_{\mathrm{m}}} - \left(\boldsymbol{D} \nabla U^h, \nabla \zeta^h\right)_{\Omega} + \left(I^{\mathrm{v}}, \zeta^h\right)_{\Omega} + \left\langle I_{\mathrm{i}}^{\mathrm{s}} + I_{\mathrm{e}}^{\mathrm{s}}, \zeta^h\right\rangle_{\Gamma_{\mathrm{m}}}, \tag{S19c}$$

for all $\varphi^h, \phi^h \in \mathcal{V}_{\mathrm{m}}^h$ and $\zeta^h \in \mathcal{S}^h$.

Taking the discrete gating variables $\boldsymbol{w}^h$ to belong to the space $\mathcal{V}_{\mathrm{m}}^h$ as for V^h, the weak problem for the ionic model (S11a) reads: find $\boldsymbol{w}^h \in \mathcal{V}_{\mathrm{m}}^h$ such that

$$\left(\dot{\boldsymbol{w}}^h, \varphi^h\right)_{\Omega_{\mathrm{m}}} = \left(\boldsymbol{g}\left(V^h, \boldsymbol{w}^h\right), \varphi^h\right)_{\Omega_{\mathrm{m}}}, \tag{S20}$$

for all $\varphi^h \in \mathcal{V}_{\mathrm{m}}^h$.

Denoting by $\mathcal{T}^h$ the triangulation of $\bar{\Omega}$, by $\mathcal{T}_{\mathrm{m}}^h \subset \mathcal{T}^h$ the triangulation of $\bar{\Omega}_{\mathrm{m}}$, and by $\mathcal{T}_{\mathrm{b}}^h \subset \mathcal{T}^h$ the triangulation of $\bar{\Omega}_{\mathrm{b}}$, we take

$$\mathcal{V}_{\mathrm{m}}^h = \left\{ v \in C^0\left(\bar{\Omega}_{\mathrm{m}}\right) : v^h\Big|_K \in \mathcal{P}^1(K) \ \forall K \in \mathcal{T}_{\mathrm{m}}^h \right\}, \tag{S21}$$

and

$$\mathcal{S}^h = \left\{ v \in C^0\left(\bar{\Omega}\right) : v^h\Big|_K \in \mathcal{P}^1\left(K\right)\ \forall K \in \mathcal{T}^h \right\}. \tag{S22}$$

In other words, we use piecewise linear interpolation for all variables. Denote by $\mathcal{N}_V = \dim \mathcal{V}_{\mathrm{m}}^h$ and $\mathcal{N}_U = \dim \mathcal{S}^h$, such that $\mathcal{N}_V \leq \mathcal{N}_U$ (the equality holds only for the case of no bath). The solution fields are approximated as

$$V^h = \sum_{A=1}^{\mathcal{N}_V} N_A \mathsf{V}_A, \quad Q^h = \sum_{A=1}^{\mathcal{N}_V} N_A \mathsf{Q}_A, \quad U^h = \sum_{C=1}^{\mathcal{N}_U} N_C \mathsf{U}_C, \quad \boldsymbol{w}^h = \sum_{A=1}^{\mathcal{N}_V} N_A \mathbf{w}_A. \tag{S23}$$

The matrix system including the ionic model becomes

$$\mathbf{M}\dot{\mathsf{V}} = \mathbf{M}\mathsf{Q}, \tag{S24a}$$

$$0 = -\mathbf{K}_{\mathrm{i}}\mathsf{V} - \tilde{\mathbf{K}}_{\mathrm{i}}\mathsf{U} - \chi C_{\mathrm{m}}\mathbf{M}\mathsf{Q} - \chi \mathsf{I}_{\mathrm{ion}} + \mathsf{I}_{\mathrm{i}}^{\mathrm{s}}, \tag{S24b}$$

$$0 = -\mathbf{K}_{\mathrm{i}}\mathsf{V} - \mathbf{K}\mathsf{U} + \mathsf{I}^{\mathrm{v}} + \mathsf{I}_{\mathrm{i}}^{\mathrm{s}} + \mathsf{I}_{\mathrm{e}}^{\mathrm{s}}, \tag{S24c}$$

$$\mathbf{S}\dot{\mathbf{w}} = \mathbf{F}\left(\mathbf{w}, \mathsf{V}\right), \tag{S24d}$$

in which

$$\left[\mathbf{K}_{\mathrm{i},AB}\right] = \left(\boldsymbol{D}_{\mathrm{i}}\nabla N_A, \nabla N_B\right)_{\Omega_{\mathrm{m}}}, \tag{S25a}$$

$$\left[\mathbf{M}_{AB}\right] = \left(N_A, N_B\right)_{\Omega_{\mathrm{m}}}, \tag{S25b}$$

$$\left[\mathbf{S}_{AmBn}\right] = \left(N_A \boldsymbol{e}_m, N_B \boldsymbol{e}_n\right)_{\Omega_{\mathrm{m}}} \tag{S25c}$$

$$\left[\mathbf{K}_{CD}\right] = \left(\boldsymbol{D}\nabla N_C, \nabla N_D\right)_{\Omega}, \tag{S25d}$$

$$\left[\tilde{\mathbf{K}}_{i,AD}\right] = \left(\boldsymbol{D}_{\mathrm{i}}\nabla N_A, \nabla N_D\right)_{\Omega_{\mathrm{m}}}. \tag{S25e}$$

The evaluation of the gating variables and the ionic current is done using nodal interpolation such that

$$\left[\mathsf{I}_{\mathrm{ion},B}\right] = \left(I_{\mathrm{ion}}\left(\mathsf{V}, \mathbf{w}\right), N_B\right)_{\Omega_{\mathrm{m}}} \approx \left(\sum_A I_{\mathrm{ion},A} N_A, N_B\right)_{\Omega_{\mathrm{m}}}, \tag{S26a}$$

$$\left[\mathbf{F}_{Bn}\right] = \left(\boldsymbol{g}\left(\mathsf{V}, \mathbf{w}\right), N_B \boldsymbol{e}_n\right)_{\Omega_{\mathrm{m}}} \approx \left(\sum_A \boldsymbol{g}_A N_A \boldsymbol{e}_m, N_B \boldsymbol{e}_n\right)_{\Omega_{\mathrm{m}}}. \tag{S26b}$$

A similar interpolation is used for the applied currents.

We refer to Pezzuto et al. (2016), and the references therein, for the analysis of the space-discretization error for cardiac electrophysiology using the finite element method.

4 TIME DISCRETIZATION

Consider the generic system

$$\partial_t u = f\left(u\right) + g\left(u\right). \tag{S27}$$

Typically, g represents the non-stiff part of the equation that can be treated explicitly, while f is a stiff term requiring implicit integration. Because of the nonlinearities of the ionic models, in cardiac electrophysiology is common to treat explicitly the reaction terms defined by the ionic currents. The linear diffusion term is usually treated implicitly. Therefore, here g represents the contribution to the equation from the ionic currents, while f represents the linear diffusion term. A general p-step IMEX method (IMplicit-EXplicit method) is written as

$$u^{n+1} = \sum_{j=0}^{p} a^j u^{n-j} + \sum_{j=-1}^{p} \Delta t b^j f^{n-j} + \sum_{j=0}^{p} \Delta t c^j g^{n-j}, \tag{S28}$$

where we used the notation $u^n = u\left(t_n\right), f^n = f\left(u^n\right)$ and $g^n = g\left(u^n\right)$. We shall consider the first-order one step IMEX BDF 1 method,

$$u^{n+1} = u^n + \Delta t\, f^{n+1} + \Delta t\, g^n, \tag{S29}$$

where the implicit term is computed with backward Euler and the explicit term with forward Euler. For the second-order, method we shall use the two step IMEX BDF 2,

$$u^{n+1} = \frac{4}{3}u^n - \frac{1}{3}u^{n-1} + \frac{2}{3}\Delta t\, f^{n+1} + \frac{2}{3}\Delta t\, \left(2g^n - g^{n-1}\right), \tag{S30}$$

where we use the BDF2 method for the implicit part and the extrapolation $g^{n+1} \approx 2g^n - g^{n-1}$for the explicit term. Alternatively we may use the IMEX BDF 2 form

$$\frac{3}{2\Delta t}\left(u^{n+1} - \frac{4}{3}u^n + \frac{1}{3}u^{n-1}\right) = f^{n+1} + 2g^n - g^{n-1}. \tag{S31}$$

Therefore, the ionic current at step n are evaluated as

$$I_{\text{ion}}^n = I_{\text{ion}}\left(V^n, \boldsymbol{w}^{n+1}\right), \tag{S32}$$

REMARK. *The final linear system deriving from the above discretization of the bidomain model for both IMEX BDF 1 and IMEX BDF 2 method can be written as*

$$\left[\begin{array}{c|cc|c} \mathsf{I} & -c\Delta t\mathsf{I} & 0 & 0 \\ \hline 0 & \beta\mathbf{M} + c\Delta t\mathbf{K}_i & \tilde{\mathbf{K}}_i & 0 \\ 0 & c\Delta t\mathbf{K}_i & \mathbf{K} & 0 \\ \hline 0 & 0 & 0 & \mathsf{I} \end{array}\right] \left\{\begin{array}{c} \mathsf{V}^{n+1} \\ \mathsf{Q}^{n+1} \\ \mathsf{U}^{n+1} \\ \mathbf{w}^{n+1} \end{array}\right\} = \left\{\begin{array}{c} \mathsf{V}^{*} \\ -\mathbf{K}_i\mathsf{V}^{*} - \mathsf{J}_i^{**} \\ -\mathbf{K}_i\mathsf{V}^{*} + \mathsf{J}_e^{**} \\ \mathbf{w}^{*} + c\Delta t\mathbf{F}^{**} \end{array}\right\}, \tag{S33}$$

with $c = 1$, $\mathsf{Y}^* = \mathsf{Y}^n$, $\mathsf{Y}^{**} = \mathsf{Y}^n$ *for IMEX BDF 1 and* $c = \frac{2}{3}$, $\mathsf{Y}^* = \frac{4}{3}\mathsf{Y}^n - \frac{1}{3}\mathsf{Y}^{n-1}$, $\mathsf{Y}^{**} = 2\mathsf{Y}^n - \mathsf{Y}^{n-1}$ *for IMEX BDF 2, with* $\mathsf{Y}^* = \{\mathsf{V}^*, \mathsf{Q}^*\}$ *and* $\mathsf{Y}^{**} = \{\mathsf{J}^{**}, \mathsf{J}_e^{**}, \mathbf{F}^{**}\}$.

PROOF. **IMEX BDF 1**: Using nodal quadrature for the ODEs system corresponding to $\mathbf{w}$ we find

$$\mathsf{V}^{n+1} = \mathsf{V}^n + \Delta t \mathsf{Q}^{n+1}, \tag{S34a}$$

$$\chi C_\mathrm{m} \mathbf{M} \mathsf{Q}^{n+1} + \tilde{\mathbf{K}}_\mathrm{i} \mathsf{U}^{n+1} = -\chi {\mathsf{I}_\mathrm{ion}}^n + \mathsf{I}_\mathrm{i}^\mathrm{s}, \tag{S34b}$$

$$\mathbf{K}_\mathrm{i} \mathsf{V}^{n+1} + \mathbf{K} \mathsf{U}^{n+1} = -\mathsf{I}_\mathrm{total}^\mathrm{v} + \mathsf{I}_\mathrm{i}^\mathrm{s} + \mathsf{I}_\mathrm{e}^\mathrm{s}, \tag{S34c}$$

$$\mathbf{w}^{n+1} = \mathbf{w}^n + \Delta t \mathbf{F}^n. \tag{S34d}$$

Using now the first equation to eliminate V^{n+1} in the second and third equations,

$$\mathsf{V}^{n+1} = \mathsf{V}^n + \Delta t \mathsf{Q}^{n+1}, \tag{S35a}$$

$$\left[\chi C_\mathrm{m} \mathbf{M} + \Delta t \mathbf{K}_\mathrm{i}\right] \mathsf{Q}^{n+1} + \tilde{\mathbf{K}}_\mathrm{i} \mathsf{U}^{n+1} = -\mathbf{K}_\mathrm{i} \mathsf{V}^n - \chi {\mathsf{I}_\mathrm{ion}}^n + \mathsf{I}_\mathrm{i}^\mathrm{s}, \tag{S35b}$$

$$\left[\Delta t \mathbf{K}_\mathrm{i}\right] \mathsf{Q}^{n+1} + \mathbf{K} \mathsf{U}^{n+1} = -\mathbf{K}_\mathrm{i} \mathsf{V}^n - \mathsf{I}_\mathrm{total}^\mathrm{v} + \mathsf{I}_\mathrm{i}^\mathrm{s} + \mathsf{I}_\mathrm{e}^\mathrm{s}, \tag{S35c}$$

$$\mathbf{w}^{n+1} = \mathbf{w}^n + \Delta t \mathbf{F}^n. \tag{S35d}$$

Using the shorthand notation, $\beta = \chi C_\mathrm{m}$, $\mathsf{J}_\mathrm{i}^n = \chi {\mathsf{I}_\mathrm{ion}}^n + \left(\mathsf{I}_\mathrm{i}^\mathrm{s}\right)^{n+1}$ and $\mathsf{J}_\mathrm{e}^n = -\left(\mathsf{I}_\mathrm{total}^\mathrm{v}\right)^{n+1} + \left(\mathsf{I}_\mathrm{i}^\mathrm{s}\right)^{n+1} + \left(\mathsf{I}_\mathrm{e}^\mathrm{s}\right)^{n+1}$ we write the system as

$$\mathsf{V}^{n+1} = \mathsf{V}^n + \Delta t \mathsf{Q}^{n+1}, \tag{S36a}$$

$$\left[\beta \mathbf{M} + \Delta t \mathbf{K}_\mathrm{i}\right] \mathsf{Q}^{n+1} + \tilde{\mathbf{K}}_\mathrm{i} \mathsf{U}^{n+1} = -\mathbf{K}_\mathrm{i} \mathsf{V}^n - \mathsf{J}_\mathrm{i}^n, \tag{S36b}$$

$$\left[\Delta t \mathbf{K}_\mathrm{i}\right] \mathsf{Q}^{n+1} + \mathbf{K} \mathsf{U}^{n+1} = -\mathbf{K}_\mathrm{i} \mathsf{V}^n + \mathsf{J}_\mathrm{e}^n, \tag{S36c}$$

$$\mathbf{w}^{n+1} = \mathbf{w}^n + \Delta t \mathbf{F}^n. \tag{S36d}$$

IMEX BDF 2: Using the IMEX BDF 2 formulas and proceeding similarly to the IMEX BDF 1 case we find

$$\mathsf{V}^{n+1} = \frac{4}{3} \mathsf{V}^n - \frac{1}{3} \mathsf{V}^n + c \Delta t \mathsf{Q}^{n+1}, \tag{S37a}$$

$$\left[\beta \mathbf{M} + c \Delta t \mathbf{K}_\mathrm{i}\right] \mathsf{Q}^{n+1} + \tilde{\mathbf{K}}_\mathrm{i} \mathsf{U}^{n+1} = -\mathbf{K}_\mathrm{i} \left(\frac{4}{3} \mathsf{V}^n - \frac{1}{3} \mathsf{V}^{n-1}\right) - \left(2\mathsf{J}^n - \mathsf{J}^{n-1}\right), \tag{S37b}$$

$$\left[c \Delta t \mathbf{K}_\mathrm{i}\right] \mathsf{Q}^{n+1} + \mathbf{K} \mathsf{U}^{n+1} = -\mathbf{K}_\mathrm{i} \left(\frac{4}{3} \mathsf{V}^n - \frac{1}{3} \mathsf{V}^{n-1}\right) + \left(2\mathsf{J}_\mathrm{e}^n - \mathsf{J}_\mathrm{e}^{n-1}\right), \tag{S37c}$$

$$\mathbf{w}^{n+1} = \mathbf{w}^n + \frac{2}{3} \Delta t \left(2\mathbf{F}^n - \mathbf{F}^{n-1}\right). \tag{S37d}$$

The above schemes are only conditionally stable. The choice of the timestep follows the CFL-like condition $\Delta t < h/v_\mathrm{f}$, as given in Rossi and Griffith (2017a), where h represents the mesh size and v_f represents the conduction velocity in the fiber (longitudinal) direction. Although this choice may not be appropriate for all choices of the ionic model (S9), in many practical cases the timestep derived by the CFL conditions above is usually smaller than the one required to guarantee temporal stability. This is because very fine spatial resolution ($h < 100\mu$m) is needed to correctly capture the sharp wavefront. For a detailed

error analysis of the temporal schemes described in this section, we refer to Ethier (2008); Roy et al. (2017), and the references therein.

5 ALGORITHMIC IMPLEMENTATION

The block structure of equation (S33) indicates that only the variables Q^{n+1} and U^{n+1} need to be solved as a unique block. In fact, given equations (S36a) and (S37a), the transmembrane potential V^{n+1} can be explicitly updated once Q^{n+1} is known. Given the block structure of equation (S33), it is possible to solve the system in 3 steps:

1. Solve the ionic model
$$\mathbf{w}^{n+1} = \mathbf{w}^{*} + c\Delta t\mathbf{F}^{**}, \tag{S38}$$
at each node and evaluate the currents J^{**} and $\mathsf{J}_{\mathrm{e}}^{**}$ using the updated values of $\mathbf{w}$ as defined in (S32).
2. Solve the linear system
$$\begin{bmatrix} \beta\mathbf{M} + c\Delta t\mathbf{K}_{\mathrm{i}} & \tilde{\mathbf{K}}_{\mathrm{i}} \\ c\Delta t\mathbf{K}_{\mathrm{i}} & \mathbf{K} \end{bmatrix} \begin{Bmatrix} \mathsf{Q}^{n+1} \\ \mathsf{U}^{n+1} \end{Bmatrix} = \begin{Bmatrix} -\mathbf{K}_{\mathrm{i}}\mathsf{V}^{*} - \mathsf{J}_{\mathrm{i}}^{**} \\ -\mathbf{K}_{\mathrm{i}}\mathsf{V}^{*} + \mathsf{J}_{\mathrm{e}}^{**} \end{Bmatrix}. \tag{S39}$$
Symmetrizing the system such that
$$\begin{bmatrix} c\Delta t\beta\mathbf{M} + c^{2}\Delta t^{2}\mathbf{K}_{\mathrm{i}} & c\Delta t\tilde{\mathbf{K}}_{\mathrm{i}} \\ c\Delta t\mathbf{K}_{\mathrm{i}} & \mathbf{K} \end{bmatrix} \begin{Bmatrix} \mathsf{Q}^{n+1} \\ \mathsf{U}^{n+1} \end{Bmatrix} = \begin{Bmatrix} -\mathbf{K}_{\mathrm{i}}\mathsf{V}^{*} - \mathsf{J}_{\mathrm{i}}^{**} \\ -\mathbf{K}_{\mathrm{i}}\mathsf{V}^{*} + \mathsf{J}_{\mathrm{e}}^{**} \end{Bmatrix}, \tag{S40}$$
we solve this block system using FGMRES (up to 1e-6 relative tol) with Block Gauss-Seidel preconditionerBrown et al. (2012); Balay et al. (1997, 2017), in which each sub-block used AMG preconditioners from H*hypre* (Falgout and Yang, 2002; Falgout et al., 2010). Specifically, system (S39) is solved using the PETSc command-line:
```
-bidomain_ksp_type fgmres -bidomain_ksp_rtol 1e-6
-bidomain_pc_type fieldsplit
-bidomain_fieldsplit_v_pc_type hypre
-bidomain_fieldsplit_ve_pc_type hypre
```
in which the `v` field represents the degrees of freedom associated with Q and `ve` represents those associated with U.
3. Update $\mathsf{V}^{n+1} = \mathsf{V}^{*} + c\Delta t\mathsf{Q}^{n+1}$.

Note that it would be possible to solve for V^{n+1}, Q^{n+1}, and U^{n+1} at once in Step 2. Since the best solution strategy would be to use backward substitution of Q^{n+1} in the equations for V^{n+1}, the resulting algorithm would be identical to the one presented here. Assembling a global matrix for the variables V^{n+1}, Q^{n+1}, and Y^{n+1} would only increase the memory used by the algorithm.

6 BATH-SIZE AND CURVATURE WITH CUBIC REACTIONS

The choice of the ionic model can influence the rate of convergence of the numerical scheme. In fact, many ionic models in the literature include discontinuous equations that may prevent numerical schemes to

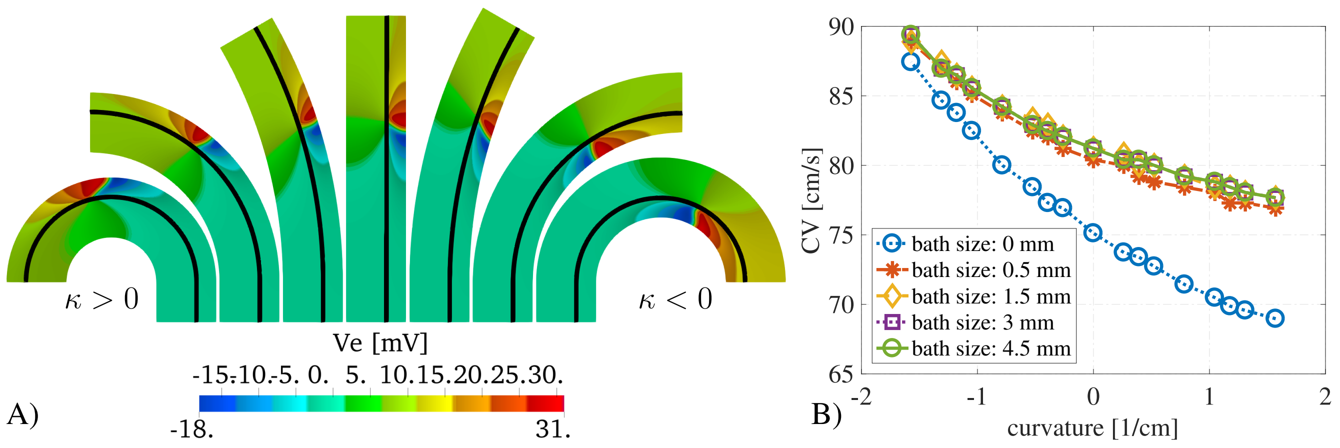

Figure S1. A) The extracellular potential on selected curvatures for the bath thickness of 3 mm. Inside the muscle, the rapid change between positive (red) and negative (blue) values of V_e locate the wave-front. In the bath, the solution does not have large gradients. B) Endocardial conduction velocities for varying bath thicknesses and muscle thickness of ℓ_m =1.5 mm with the cubic reaction model.

converge with their optimal rate (Arthurs et al., 2012). As already shown by Rossi and Griffith (2017b), a simple cubic reaction term can give second-order convergence rates. Therefore, we set the ionic current to be given asPezzuto et al. (2016)

$$I_{\text{ion}} = I_{\text{ion}}(V) = k(V - V_0)(V - V_1)(V - V_2), \tag{S41}$$

in which V_0, V_1 and V_2 are the resting, threshold and depolarization potentials, and k is a parameter controlling the steepness of the wavefront. In the following tests, we set $k = 1400$, $V_0 = -85$ mV, $V_1 = -57.6$ mV and $V_2 = 30$ mV. Although this simple reaction term cannot reproduce the repolarization of the cardiac cells, it can be used as an idealized model of the wave-front propagation. Here, we fix the muscle size to $\ell_m = 1.5$ mm and we consider baths of size $\ell_b = 0, 0.05, 0.15, 0.3$ and 0.4 cm, corresponding to the bath-tissue thickness ratios, 0:1, 1:3, 1:1, 2:1, 3:1.

We show in Fig. S1A, the extracellular potential and the bath potential at $t = 20$ ms computed using a bath thickness of 3 mm for some of the tested curvatures. The sharp change in sign of the extracellular potential (passing from red to blue) in the tissue region localizes the wave-front. We can see from Fig. S1A that the wavefront has different shapes at different curvatures.

The resulting conduction velocities, shown in Fig. S1B, demonstrate the dependence on the domain curvature. Fig. S1B shows that the size of the bath together with curvature influence the measured conduction velocities.

Finally, we show in Fig. S2 the bipolar signals registered on the endocardial surface Γ_i for several bath thicknesses. The perfusing bath reduces the amplitude of the signal which reaches an amplitude of about 12 mV for bath thickness greater than 0.15 cm. The effects of the curvature on the bipolar signal are small and we show the signals only for the straight case and for the largest curvature magnitudes.

REFERENCES

Rossi S, Griffith BE. Incorporating inductances in tissue-scale models of cardiac electrophysiology. *Chaos: An Interdisciplinary Journal of Nonlinear Science* **27** (2017a) 093926. doi:10.1063/1.5000706.

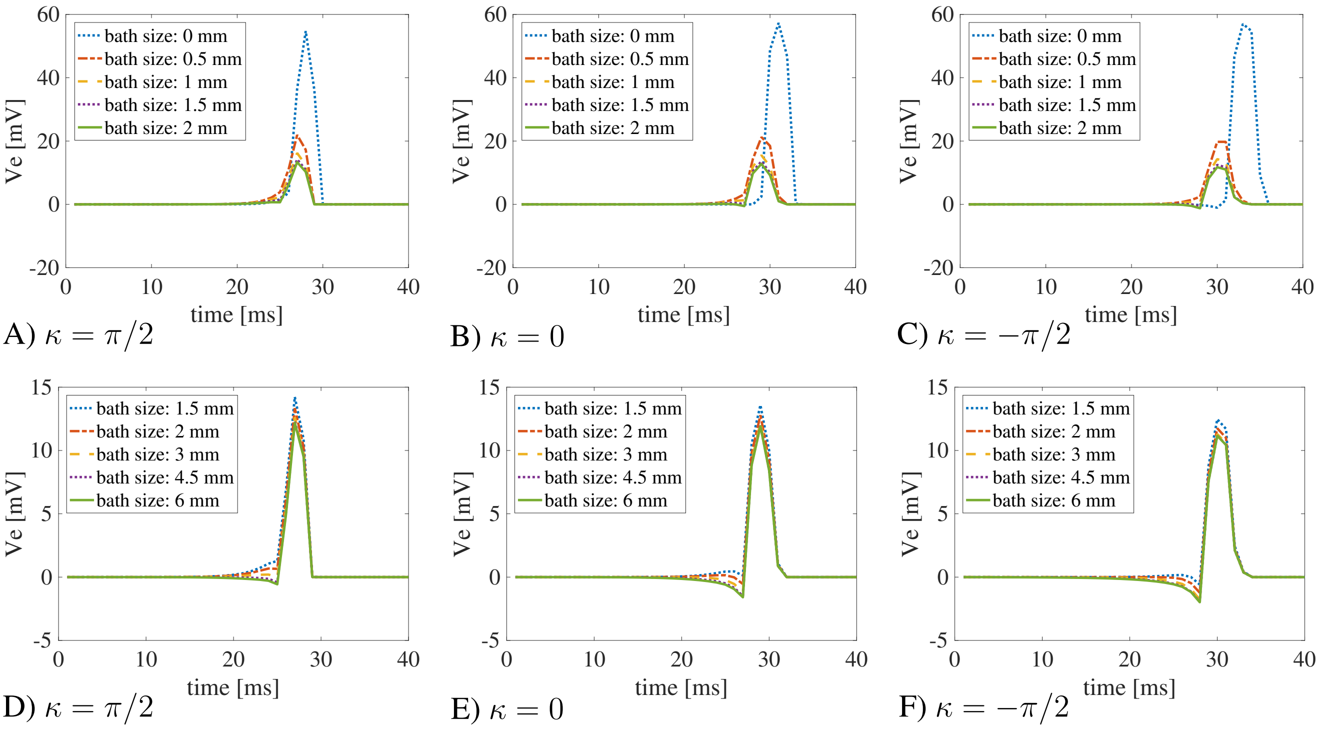

Figure S2. Bipolar signals $V_e^2 - V_e^1$ recorded at 1 kHz for three selected curvatures $\kappa = \pi/2\ \mathrm{cm}^{-1}, 0\ \mathrm{cm}, \pi/2\ \mathrm{cm}^{-1})$ for different bath sizes and muscle thickness $\ell_\mathrm{m} = 1.5$ mm using the cubic reaction model. A,B,C) Bipolar signals for bath sizes between 0-2 mm. D,E,F) Bipolar signals for bath sizes between 1.5-6 mm. An overlap of the data has been used between the top and bottom rows to better understand the differences in signals for different bath sizes. The curvature of the domain does not play a major role in the recorded signals. Large differences in the signal amplitudes can be found for bath sizes smaller than 2 mm. Although minor differences can also be noted for bath larger than 1.5mm, the amplitude of the signals is well captured for baths of size at least 3 mm.

Henriquez CS, Muzikant AL, Smoak CK. Anisotropy, fiber curvature, and bath loading effects on activation in thin and thick cardiac tissue preparations. *Journal of cardiovascular electrophysiology* **7** (1996) 424–444.

Keener JP, Sneyd J. *Mathematical physiology*, vol. 1 (Springer) (1998).

Griffith BE, Peskin CS. Electrophysiology. *Communications on Pure and Applied Mathematics* **66** (2013) 1837–1913.

Franzone PC, Pavarino LF, Scacchi S. *Mathematical cardiac electrophysiology*, vol. 13 (Springer) (2014).

Vigmond E, Dos Santos RW, Prassl A, Deo M, Plank G. Solvers for the cardiac bidomain equations. *Progress in biophysics and molecular biology* **96** (2008) 3–18.

Plank G, Leon LJ, Kimber S, Vigmond EJ. Defibrillation depends on conductivity fluctuations and the degree of disorganization in reentry patterns. *Journal of cardiovascular electrophysiology* **16** (2005) 205–216.

Franzone PC, Deuflhard P, Erdmann B, Lang J, Pavarino LF. Adaptivity in space and time for reaction-diffusion systems in electrocardiology. *SIAM Journal on Scientific Computing* **28** (2006) 942–962.

Seemann G, Höper C, Sachse FB, Dössel O, Holden AV, Zhang H. Heterogeneous three-dimensional anatomical and electrophysiological model of human atria. *Philosophical Transactions of the Royal Society of London A: Mathematical, Physical and Engineering Sciences* **364** (2006) 1465–1481.

Pathmanathan P, Bernabeu MO, Bordas R, Cooper J, Garny A, Pitt-Francis JM, et al. A numerical guide to the solution of the bidomain equations of cardiac electrophysiology. *Progress in Biophysics and Molecular Biology* **102** (2010) 136–155. doi:10.1016/j.pbiomolbio.2010.05.006.

Bishop MJ, Plank G. Representing cardiac bidomain bath-loading effects by an augmented monodomain approach: application to complex ventricular models. *IEEE Transactions on Biomedical Engineering* **58** (2011) 1066–1075.

Landajuela M, Vergara C, Gerbi A, Dedé L, Formaggia L, Quarteroni A. Numerical approximation of the electromechanical coupling in the left ventricle with inclusion of the purkinje network. *International journal for numerical methods in biomedical engineering* (2018) e2984.

Bendahmane M, Chamakuri N. Numerical analysis for an optimal control of bidomain-bath model. *Journal of Differential Equations* **263** (2017) 2419–2456.

Pezzuto S, Hake J, Sundnes J. Space-discretization error analysis and stabilization schemes for conduction velocity in cardiac electrophysiology. *International journal for numerical methods in biomedical engineering* **32** (2016).

Ethier Y Marc; Bourgault. Semi-implicit time-discretization schemes for the bidomain model. *SIAM Journal on Numerical Analysis* **46** (2008). doi:10.1137/070680503.

Roy T, Bourgault Y, Pierre C. Analysis of time-stepping methods for the monodomain model. *arXiv preprint arXiv:1710.01106* (2017).

Brown J, Knepley MG, May DA, McInnes LC, Smith B. Composable linear solvers for multiphysics. *Parallel and Distributed Computing (ISPDC), 2012 11th International Symposium on* (IEEE) (2012), 55–62.

Balay S, Gropp WD, McInnes LC, Smith BF. Efficient management of parallelism in object oriented numerical software libraries. Arge E, Bruaset AM, Langtangen HP, editors, *Modern Software Tools in Scientific Computing* (Birkhäuser Press) (1997), 163–202.

Balay S, Abhyankar S, Adams MF, Brown J, Brune P, Buschelman K, et al. PETSc users manual. Tech. Rep. ANL-95/11 - Revision 3.8, Argonne National Laboratory (2017).

Falgout RD, Yang UM. hypre: A library of high performance preconditioners. *International Conference on Computational Science* (Springer) (2002), 632–641.

Falgout R, Cleary A, Jones J, Chow E, Henson V, Baldwin C, et al. Hypre: High performance preconditioners. *Users Manual. Version* **1** (2010).

Arthurs CJ, Bishop MJ, Kay D. Efficient simulation of cardiac electrical propagation using high order finite elements. *J. Comput. Physics* **231** (2012) 3946–3962. doi:10.1016/j.jcp.2012.01.037.

Rossi S, Griffith BE. Incorporating inductances in tissue-scale models of cardiac electrophysiology. *Chaos: An Interdisciplinary Journal of Nonlinear Science* **27** (2017b) 093926.
